# Supplementary material for: Analysis of genetically independent phenotypes identifies shared genetic factors associated with chronic musculoskeletal pain conditions
Source: Commun Biol. 2020 Jun 25;3:329. doi: 10.1038/s42003-020-1051-9 (PMC7316754; doi:10.1038/s42003-020-1051-9)

Supplementary Methods and Figures

Contents

Phenotype definition 2
GWAS-MAP platform 3
Genetically independent phenotypes (GIP) analysis 7
Phenotypic correlations 10
Validation of GIP method using other datasets 11
(1) Results of analysis of four simulated genetically independent traits 12
(2) Results of analysis of four simulated genetically identical traits 13
(3) Results of analysis of four closely genetically related traits 14
(4) Results of analysis of four closely genetically related traits and one trait that genetically is not strongly related to some of these traits 15
(5) Results of analysis of four traits that are not strongly genetically dependent 16
Testing for pleiotropy using SMR/HEIDI approach 18
Supplementary Figure 1 19
Supplementary Figure 2 20
Supplementary Figure 3 23
Supplementary Figure 4 25
Supplementary Figure 5 31
Supplementary Figure 6 32

Phenotype definition

Within the frame of the UK Biobank study, participants were asked to complete web-based questionnaires which included questions related to pain experience and duration.

We defined chronic pain patients and corresponding controls based on their answers to “SY5” and “SY5B” questions[1]:

Q. No	Stem	Responses	Select from
SY5	In the last month have you experienced any of the following that interfered with your usual activities?<br>(You can select more than one answer)	Select from<br>- 01 Headache<br>- 02 Facial pain<br>- 03 Neck or shoulder pain<br>- 04 Back pain<br>- 05 Stomach or abdominal pain<br>- 06 Hip pain<br>- 07 Knee pain<br>- 08 Pain all over the body<br>- NN None of the above<br>- DA Prefer not to answer	If any pain selected ask SY5B for that pain.<br>If none of the above or DA go to SY1
SY5B	Have you had *** for more than 3 months?<br><br>**** for each pain selected in SY5 insert the response from SY5 with an ‘s’ following the word pain (eg, Have you had headaches for more than 3 months? Or Have you had neck or shoulder pains for more than 3 months? Or Have you had pains all over the body for more than 3 months?)*	Select from<br>- YE Yes<br>- NO No<br>- UN Do not know<br>- DA Prefer not to answer	Loop to go through SY5B for each pain selected (except ‘pain all over the body’ where only one selection allowed in SY5.<br>Go to SY1 when all selections completed.

- Individuals who preferred not to answer the SY5 question as well as individuals who selected “Pain all over the body” AND answered “Yes” to the SY5B question were excluded from the study.
- For each studied pain phenotype (back, neck/shoulder, knee, and hip), individuals who selected the corresponding pain in the SY5 section AND answered “Yes” to the SY5B question were defines as cases.
- Similarly, individuals who did not choose a specific pain in the SY5 section (either answering “None of the above” or selecting another pain) as well as individuals who selected the studied pain AND answered “No” to the SY5B question were defined as controls to each pain phenotype.

REFERENCES:

1. UK Biobank touchscreen questionnaire. https://www.ukbiobank.ac.uk/wp-content/uploads/2011/06/Touch_screen_questionnaire.pdf?phpMyAdmin=trmKQlYdjjnQIgJ,fAzikMhEnx6

GWAS-MAP platform

GWAS-MAP platform was originally created to study cardiovascular diseases. It integrates a database of summary-level GWAS results for different complex traits, including plasma/IgG N-glycome traits, levels of circulating metabolites, cytokines, growth factors, and other proteins. In the present study, we additionally added 18 chronic musculoskeletal pain-related traits and osteoarthritis . A brief description of data is given in Table SM1. All 2,262 traits are listed in Supplementary Table 1b.

SNPs from each GWAS were matched with polymorphisms from 1000 Genomes Project Phase 3 v5 reference panel. SNPs with conflicting data on rs id, position, and alleles were excluded. For SNPs that have passed this filtering, alleles were harmonized across all GWAS and sorted in a lexicographic order.

GWAS summary statistics are stored using the ClickHouse database management system (https://clickhouse.yandex/). For each trait, we created an annotation file that contains information about the study design and key characteristics of association analysis (name of the cohort, sample size, model of inheritance, trait transformations, reference population, etc.). Metadata is organized with the PostgreSQL database system (https://www.postgresql.org/).

Besides a GWAS database, our platform contains embedded software for LD Score regression[1], 2-sample Mendelian randomization analysis[2,3], and our implementation of SMR/HEIDI analysis[4] (see below).

Table SM1. Data included in the GWAS-MAP database.

Dataset/source	Number of traits	Description		Reference
The Neale Lab	639	Complex traits from the UK Biobank*		http://www.nealelab.is/
The Gene ATLAS	34	Complex traits from the UK Biobank*		http://geneatlas.roslin.ed.ac.uk/
"Metabolomics_NMR"	123	Circulating metabolites quantified with the NMR metabolomics platform (University Hospitals of Strasbourg, France)		[5]
"Protein biomarkers_Olink"	82	Plasma proteins considered relevant to cardiovascular disease measured with the ProSeek CVD array I (Olink Biosciences, Sweden)		[6]
"Proteomics_SOMAscan"	1,124	Blood circulating proteins measured with the SOMAscan platform (SomaLogic Inc., USA)		[7]
"CAD_traits"	8	Coronary artery disease-related traits	Coronary artery disease	[8]
			Coronary artery disease	[9]
			Myocardial infarction	[10]
			Fasting glucose	[11]
			Cigarettes smoked per day	[12]
			Body mass index	[13]
			Waist-hip ratio	[14]
			Educational attainment	[15]
"IBD"	2	Inflammatory bowel disease (Crohn's disease and ulcerative colitis)		[16]
"Cytokines and growth factors"	41	Circulating cytokines and growth factors (measured in plasma or serum)		[17]
"IgG glycome"	77	Plasma IgG N-glycome traits measured by UPLC		https://doi.org/10.7488/ds/2481
"Plasma glycome"	113	Plasma N-glycome traits measured by UPLC		https://doi.org/10.5281/zenodo.1298406
**Additionally added**				
"Chronic pain"	18	Chronic pain-related traits	Chronic back, neck/shoulder, knee, hip pain (UK Biobank traits)** (two datasets – discovery dataset and European ancestry meta-analysis – for each trait)	GWAS summary statistics were obtained in the present study
			Four genetically independent phenotypes for back, neck/ shoulder, knee, and hip pain (two datasets – discovery dataset and European ancestry meta-analysis – for each phenotype)	
			Chronic stomach/abdominal pain; headache (UK Biobank traits)** (European ancestry meta-analysis)	
"Michigan PheWeb"	1	Osteoarthritis	The UK biobank trait. GWAS was performed using SAIGE method[18], and the results were deposited in the Michigan PheWeb database (trait "740: Osteoarthrosis").	http://pheweb.sph.umich.edu/SAIGE-UKB/pheno/740 [18]

* Binary traits with the number of cases or controls < 2000 were not included in the database.

- Data from the Neale Lab database were downloaded on December 15, 2017.
- Data from the Gene ATLAS were downloaded on December 8, 2017.

** Genotyping and imputation data were obtained from the UK Biobank March 2018 data release under the project #18219 "Genetic and epidemiological analyses of low back pain".

REFERENCES:

1. Bulik-Sullivan, B. K. *et al.* LD Score regression distinguishes confounding from polygenicity in genome-wide association studies. *Nat. Genet.* **47,** 291–295 (2015).
2. Hemani, G. *et al.* MR-Base: a platform for systematic causal inference across the phenome using billions of genetic associations. Preprint at https://doi.org/10.1101/078972 (2016).
3. Hemani, G. *et al.* The MR-Base platform supports systematic causal inference across the human phenome. *Elife* **7,** e34408; 10.7554/eLife.34408 (2018).
4. Zhu, Z. et al. Integration of summary data from GWAS and eQTL studies predicts complex trait gene targets. *Nat. Genet.* **48**, 481–487 (2016).
5. Kettunen, J. *et al.* Genome-wide study for circulating metabolites identifies 62 loci and reveals novel systemic effects of LPA. *Nat. Commun.* **7,** 11122; 10.1038/ncomms11122 (2016).
6. Folkersen, L. et al. Mapping of 79 loci for 83 plasma protein biomarkers in cardiovascular disease. *PLOS Genet.* **13**, e1006706; 10.1371/journal.pgen.1006706 (2017).
7. Suhre, K. et al. Connecting genetic risk to disease end points through the human blood plasma proteome. *Nat. Commun.* **8**, 14357; 10.1038/ncomms14357 (2017).
8. Schunkert, H. et al. Large-scale association analysis identifies 13 new susceptibility loci for coronary artery disease. *Nat. Genet.* **43**, 333–338 (2011).
9. Howson, J. M. M. *et al.* Fifteen new risk loci for coronary artery disease highlight arterial-wall-specific mechanisms. *Nat. Genet.* **49,** 1113–1119 (2017).
10. Nikpay, M. *et al.* A comprehensive 1000 Genomes–based genome-wide association meta-analysis of coronary artery disease. *Nat. Genet.* **47,** 1121–1130 (2015).
11. Dupuis, J. *et al.* New genetic loci implicated in fasting glucose homeostasis and their impact on type 2 diabetes risk. *Nat. Genet.* **42,** 105–116 (2010).
12. The Tobacco and Genetics Consortium. Genome-wide meta-analyses identify multiple loci associated with smoking behavior. *Nat. Genet.* **42,** 441–447 (2010).
13. Locke, A. E. *et al.* Genetic studies of body mass index yield new insights for obesity biology. *Nature* **518,** 197–206 (2015).
14. Shungin, D. *et al.* New genetic loci link adipose and insulin biology to body fat distribution. *Nature* **518,** 187–196 (2015).
15. Okbay, A. *et al.* Genome-wide association study identifies 74 loci associated with educational attainment. *Nature* **533,** 539–542 (2016).
16. de Lange, K. M. et al. Genome-wide association study implicates immune activation of multiple integrin genes in inflammatory bowel disease. *Nat. Genet.* **49**, 256–261 (2017).
17. Ahola-Olli, A. V. et al. Genome-wide association study identifies 27 loci influencing concentrations of circulating cytokines and growth factors.

Am. J. Hum. Genet. **100**, 40–50 (2017).
18. Zhou, W. et al. Efficiently controlling for case-control imbalance and sample relatedness in large-scale genetic association studies. *Nat. Genet.* **50**, 1335–1341 (2018).

Genetically independent phenotypes (GIP) analysis

To elucidate the genetic component explaining most cases of four chronic musculoskeletal pain phenotypes, we proposed to use a modified **principal component analysis** (PCA) technique. PCA is a statistical procedure that uses an orthogonal transformation to convert a set of possibly correlated variables into a set of linearly uncorrelated variables called principal components (PCs). Each PC is a linear combination of the original variables. The first PC explains as much variability as possible (has the largest possible variance), and each succeeding component accounts for the largest proportion of the remaining variability under the constraint that it is orthogonal to the preceding components. The resulting vectors of orthogonal transformation coefficients (denoted as $\boldsymbol{a_i}$) are an uncorrelated orthogonal basis set. PCA is sensitive to the relative scaling of the original variables. In the case of positive semidefinite covariance matrix of original variables ($\sum$), $\boldsymbol{a_i}$ are eigenvectors of $\sum$. Each corresponding eigenvalue is proportional to the portion of the "variance explained" (more correctly of the sum of the squared distances of the points from their multidimensional mean) that is associated with each eigenvector.

To decompose the traits of interest into the genetically independent components, we proposed to use the matrix of genetic covariances $\boldsymbol{\Omega}$ (instead of the matrix of phenotypic covariance used in conventional PCA for biological traits) for extraction of $\boldsymbol{a_i}$. We termed the resulting principal components **"genetically independent phenotypes" (GIPs)**.

GIPs have several properties:

1. Each GIP is the specific linear combination of original traits. Thus, corresponding GWAS results can be obtained for each GIP, and each GIP can be analyzed as a separate trait using *in-silico* follow-up approaches.
2. GIPs are genetically independent from each other: pairwise genetic correlations between any GIPs are zero (albeit phenotypic correlations of original traits may not be equal to zero).
3. Confidence intervals (CI) for $\boldsymbol{a_i}$ can be estimated using the standard errors of genetic covariance matrix estimation.

Technical details of the genetic principle component analysis approach are provided below.

Denote the following variables:

Ω – the matrix of genetic covariances (m x m, where m is the number of traits)

Ω_{SE} – the matrix of the standard errors of genetic covariances (m x m)

$\sum_{ph}$ – the matrix of phenotypic covariances (m x m); in the case of standardized traits it is equal to the matrix of phenotypic correlations

B – the matrix of effect sizes (β) for m phenotypes (M x m, where M is the number of SNPs in the analysis). b_i is the i-th column of B.

SE – the matrix of standard errors of β for m phenotypes (M x m). SE_i is the i-th column of SE.

$varY_i$ – the variance of the i-th trait. After standardization, $varY_i = 1$.

SD_i – the standard deviation of i-th trait. $SD_i = \sqrt{varY_i}$

B_s – the matrix of standardized β for m phenotypes (M x m). b^s_i is the i-th column of B_s.

SE_s – the matrix of standardized standard errors for m phenotypes (M x m). SE^s_i is the i-th column of SE_s.

A – the matrix of eigenvectors of Ω (m x m). Each column is a_i, $\{a_1 \ldots a_m\}$ – the vector of orthogonal transformation coefficients of m original traits into m GIPs $\{GIP_1 .. GIP_m\}$

A_s – the matrix of scaled eigenvectors of Ω (m x m). Each column is a^s_i, $\{a^s_1 \ldots a^s_m\}$ – the vector of orthogonal transformation coefficients of m original traits into m GIPs scaled to make the GIPs' variance equal to 1.

L – the vector of eigenvalues $\{l_1 \ldots l_m\}$.

We performed the following procedure to calculate GIPs for four studied pain phenotypes:

1. Estimated Ω and Ω_{SE} using LDSC software (https://github.com/bulik/ldsc/).
2. Estimated $varY_i$ and Pearson correlation matrix for four pain phenotypes.
3. Standardized GWAS summary statistics for four pain phenotypes ($\beta_i^s = \beta_i / SD_i$ and $SE_i^s = SE_i / SD_i$).
4. Checked whether all eigenvalues were positive for Ω.
5. Estimated eigenvalues (L) and the matrix of eigenvectors (A) of Ω.
6. If the coefficient of a given eigenvector for back pain was negative ($a_{i,back\ pain} < 0$), we changed the signs for all coefficients in its eigenvector ($a_i = -a_i$)
7. Estimated variance for GIP as $var(GOP_i) = \sum[(a_i \otimes a_i) \circ \Sigma_{ph}]$, where ⊗ is an outer product.
8. Scaled coefficients for GIPs as $a_i^s = a_i / SD(GOP_i)$.
9. Estimated 95% CI for a_i (see below)*.
10. Provided GWAS results for each GIP (see below)**.

*For estimation of 95% CI for GIPs, the Monte Carlo approach was used. We performed 1000 cycles of simulations. In each round, we simulated the noise component for matrix of genetic correlations Ω - the matrix Ω^{noise} (m x m). Each element i,j ($i > j$) Ω^{noise} is sampled from the normal distribution with zero mean and standard deviation equal to the i,j element of matrix of standard errors ($\Omega_{i,j}^{SE}$). The resulting covariance matrix was obtained as the sum of Ω and Ω^{noise}. Than the standardized eigenvalues were calculated as described above formulating the empirical distribution of each element of A_s matrix. For each element of A_s, 95% CI was obtained as an absolute difference between 0.975 and 0.025 quantiles divided by 2.

**Estimation of GWAS for GIPs was performed using the following procedure:

1. Effect sizes for M SNPs were calculated as $\beta_{GOP_i} = B_s \times a_i^s$, where × is an inner product.
2. Estimation of variance for GIPs was calculated as $varGOP_i = \sum[(a_i \otimes a_i) \circ \sum_{ph}]$. Phenotypic correlation matrix $\sum_{ph}$ was obtained from the specific studied population.
3. The standard errors of effect sizes for M SNPs were calculated as

   $SE_{GPC_i} = \sqrt{varGOP_i * \left({SE_1^s}^2 + \frac{{b_1^s}^2}{N}\right) - \frac{\beta_{GOP_i}^2}{N}}$, where N is the sample size.
4. Scaling of effect sizes and standard errors were performed as described above.
5. The corresponding P-values were estimated using Wald test ($Z - score = \beta_{GOP_i}/SE_{GOP_i}$).

- **The total genetic variance of m original traits explained by each GIP was calculated as $\boldsymbol{R_{GOP_i}^2 = l_i/\sum_{i=1}^{m} l_i}$.**
- The heritability of each GIP was calculated as $h_{GOP_i}^2 = \frac{\sum[(a_i \otimes a_i) \circ \Omega]}{\sum[(a_i \otimes a_i) \circ \sum_{ph}]}$
- Genetic correlations between GIPs and original traits as well as between each other were calculated as

  $$\rho_{genetic}\{c_1|c_2\} = \frac{\sum[(c_1 \otimes c_2) \circ \Omega]}{\sqrt{\sum[(c_1 \otimes c_1) \circ \Omega] \times \sum[(c_2 \otimes c_2) \circ \Omega]}}$$

  given that GIPs are linear combinations of original traits (c_1 and c_2, in case of GOP_i $c_j = a_i^s$),
- The contribution of each GIP into the genetic basis of original traits (the genetic variance explained by GIP) was estimated as squared genetic correlation coefficient of GIP with a given trait.

Phenotypic correlations

- Phenotypic correlations between pain phenotypes were estimated using R statistical software, http://www.r-project.org (function "cor(method="Pearson")").
- $\sum_{ph}$ – the matrix of phenotypic covariances (m x m); in the case of standardized traits it is equal to the matrix of phenotypic correlations.
- Predicted phenotypic correlations between GIPs and original traits as well as between each other were estimated as

$$\rho_{phenotypic}\{c_1|c_2\} = \frac{\sum\left[(c_1 \otimes c_2) \circ \sum_{ph}\right]}{\sqrt{\sum\left[(c_1 \otimes c_1) \circ \sum_{ph}\right] \times \sum\left[(c_2 \otimes c_2) \circ \sum_{ph}\right]}}$$

given that GIPs are linear combinations of original traits (c_1 and c_2, in case of $GOP_i\ c_j = a_i^s$).

Validation of GIP method using other datasets

To assess the general performance of the proposed GIP method, we tested it using five scenarios. Two of these scenarios are simulated, and three are real datasets:

(1) Simulations of four genetically independent traits (rg = 0 for each pair);

(2) Simulations of four genetically identical traits (rg = 1 for each pair);

(3) Real data on four closely genetically related anthropometric traits;

(4) Real data on four closely genetically related traits and one trait that genetically is not strongly related to some of these traits;

(5) Real data on four traits that are not strongly genetically dependent.

For each case, we present a figure depicting the matrix of genetic correlations with estimations of heritability.

(1) Results of analysis of four simulated genetically independent traits

We applied the GIP method to four simulated genetically independent traits using two matrices: the genetic covariation matrix calculated as rg_cov = I*h^2 and the phenotypic correlation matrix calculated as phe_cor = I, where I is the identity matrix, and h^2 is a scalar equal to 0.5. The results are presented in Figure SM1.

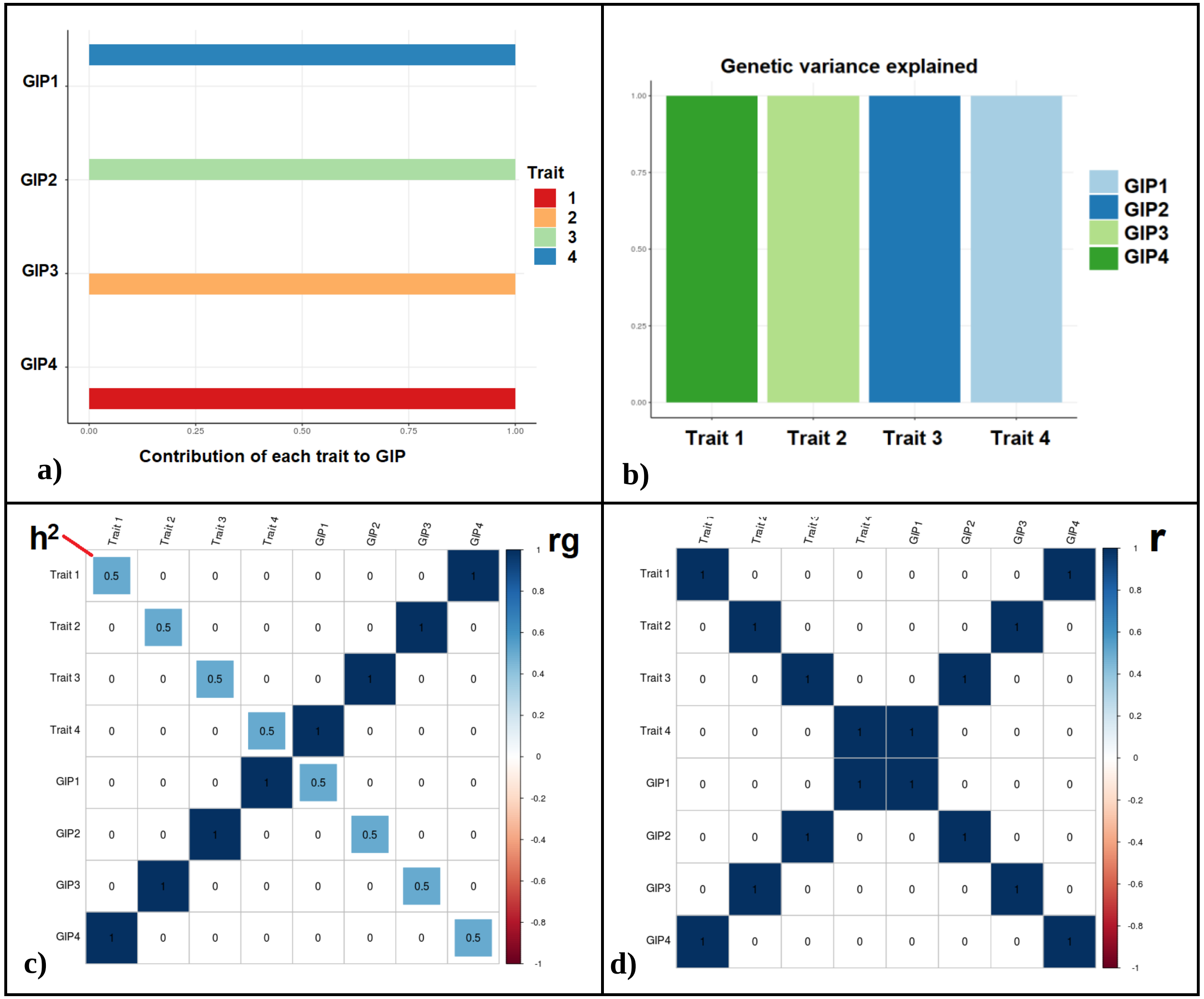

Figure SM1. Genetically independent phenotypes (GIP) for scenario (1).

a. Barplots depicting the contribution of the four original traits to each GIP. The bars represent orthogonal transformation coefficients.

b. Genetic variance of the original traits explained by four GIPs.

c. Estimated matrix of genetic correlations (rg) between the original traits and GIPs. The diagonal elements represent estimates of SNP-based heritability (h^2) for each trait.

d. Matrix of phenotypic correlations (r) between the original phenotypes and GIPs.

Conclusion

As can be clearly seen from Figure SM1b, none of GIPs represents a shared genetic background since it does not exist in the case of genetically independent traits. In this case, each GIP represents a separate trait.

(2) Results of analysis of four simulated genetically identical traits

We applied the GIP method to four simulated genetically identical traits using two matrices: the genetic covariation matrix calculated as rg_cov = J*sqrt($h^2 \otimes h^2$) and the phenotypic correlation matrix calculated as phe_cor = J, where J is the 4 x 4 matrix of ones, h^2 is a vector of four elements equal to 0.5, $\otimes$ is an outer product, and sqrt is a square root. The results are presented in Figure SM2.

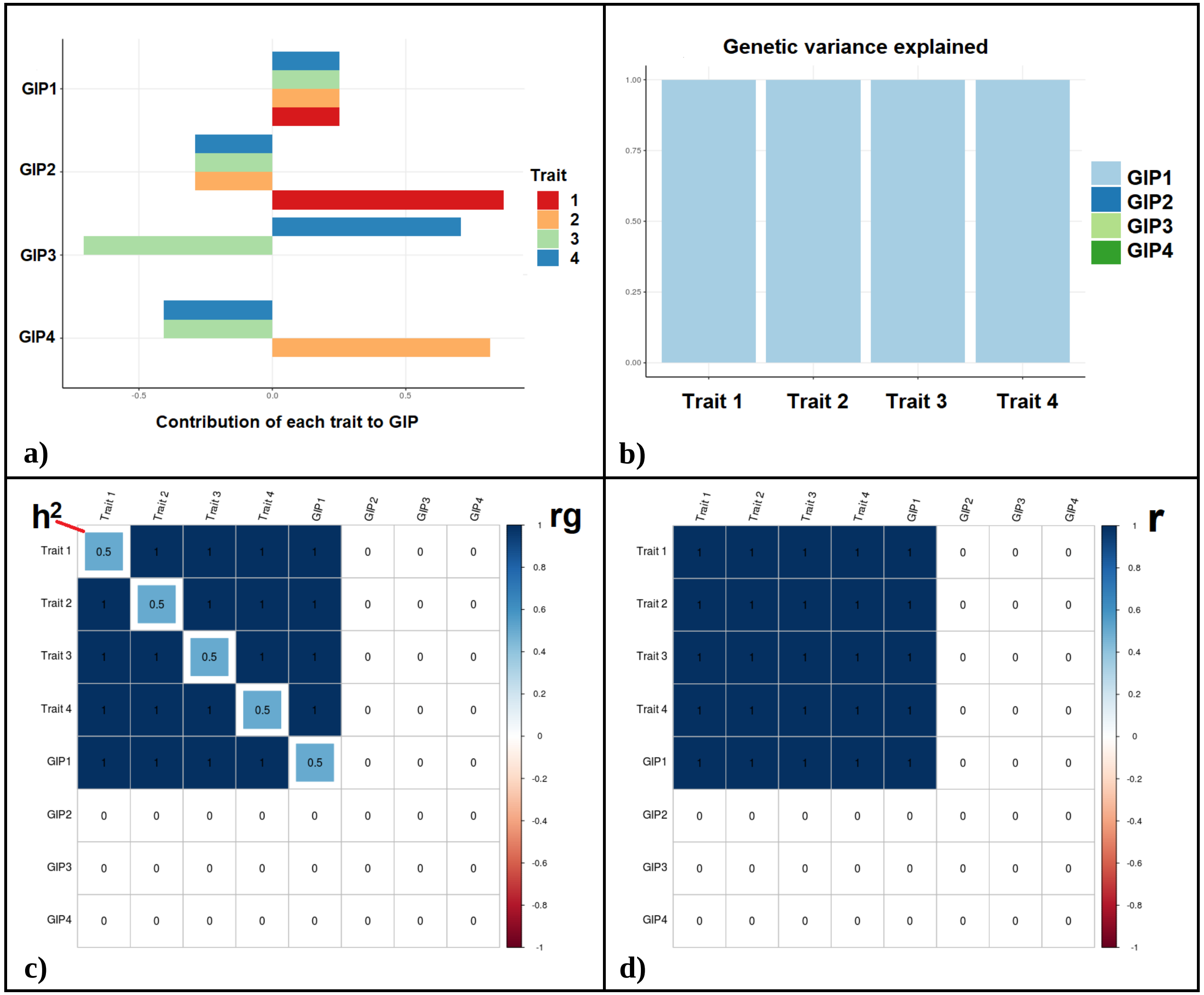

Figure SM2. Genetically independent phenotypes (GIP) for scenario (2).

a. Barplots depicting the contribution of the four original traits to each GIP. The bars represent orthogonal transformation coefficients.

b. Genetic variance of the original traits explained by four GIPs.

c. Estimated matrix of genetic correlations (rg) between the original traits and GIPs. The diagonal elements represent estimates of SNP-based heritability (h^2) for each trait.

d. Matrix of phenotypic correlations (r) between the original phenotypes and GIPs.

Conclusion

As can be seen from Figure SM2c, the only GIP with non-zero heritability is the first GIP (GIP1). This GIP explains 100% of the genetic variance of the studied traits (Figure SM2b). Given that in this extreme scenario all the original traits are genetically identical, their shared genetic background can be defined as the genetic background of one of the traits. GIP1 is produced by the GIP method as the sum of four traits with equal contribution that is mathematically equal to any of the original traits.

(3) Results of analysis of four closely genetically related traits

To assess the performance of the GIP method on real data, we applied it to four closely genetically related anthropometric traits: body mass index (BMI); weight; hip circumference (HC); waist circumference (WC). GWAS results for all traits were downloaded from The Neale Lab database (http://www.nealelab.is/uk-biobank) (GWAS summary statistics; the UK Biobank study participants; N = 336, 000). The results are presented in Figure SM3.

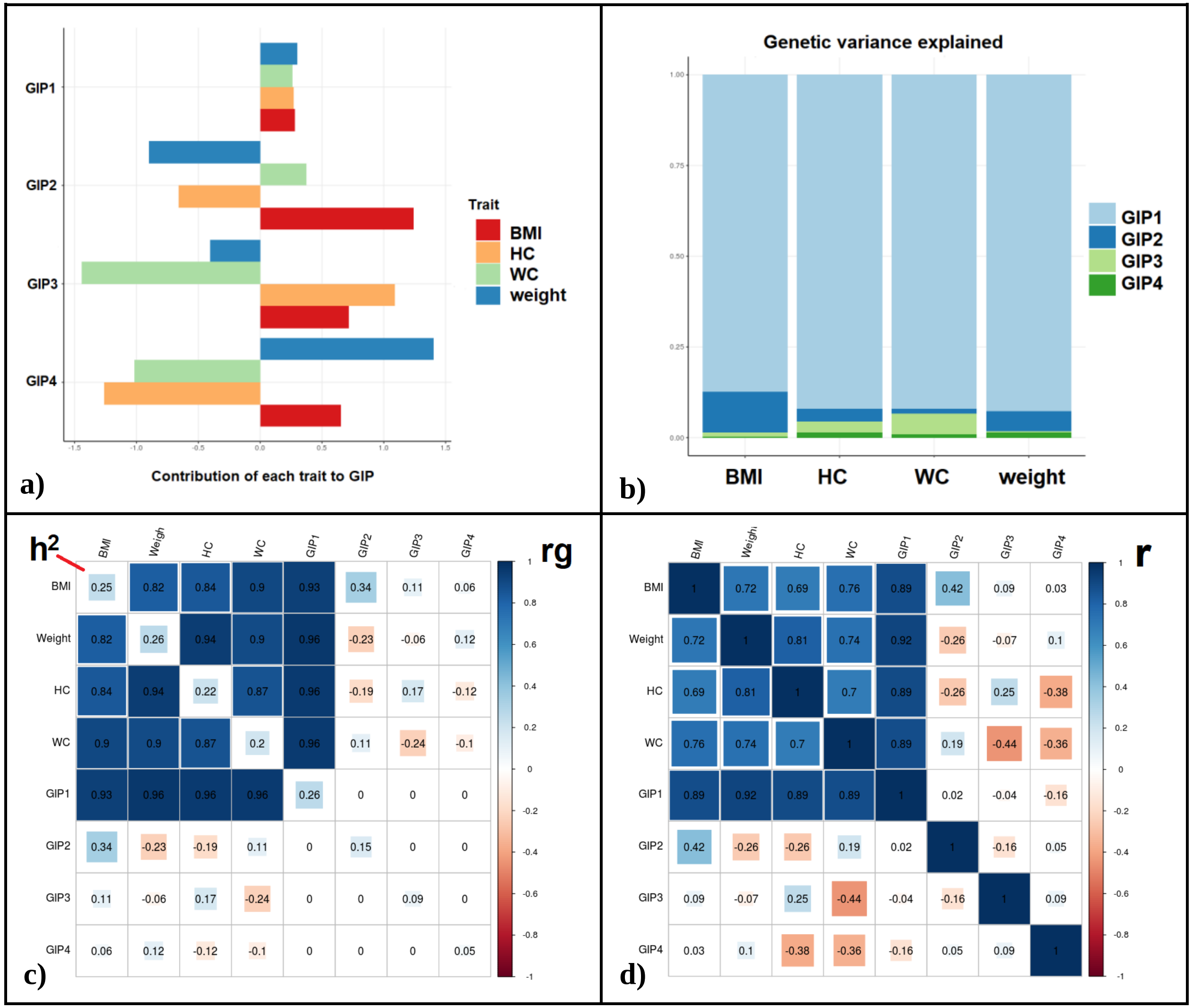

Figure SM3. Genetically independent phenotypes (GIP) for scenario (3).

a. Barplots depicting the contribution of the four original traits to each GIP. The bars represent orthogonal transformation coefficients. BMI, body mass index; HC, hip circumference; WC, waist circumference.

b. Genetic variance of the original traits explained by four GIPs.

c. Estimated matrix of genetic correlations (rg) between the original traits and GIPs. The diagonal elements represent estimates of SNP-based heritability (h^2) for each trait.

d. Matrix of phenotypic correlations (r) between the original phenotypes and GIPs.

Conclusion

As can be seen from Figure SM3b, GIP1 explains the largest proportion of the genetic variance of all studied anthropometric traits and therefore approximates their shared genetic background.

(4) Results of analysis of four closely genetically related traits and one trait that genetically is not strongly related to some of these traits

As a next step, we added height to the group of four genetically related traits considered in scenario (3). The results are presented in Figure SM4. Height has the highest heritability and is weakly genetically correlated with BMI and MC (Figure SM4c).

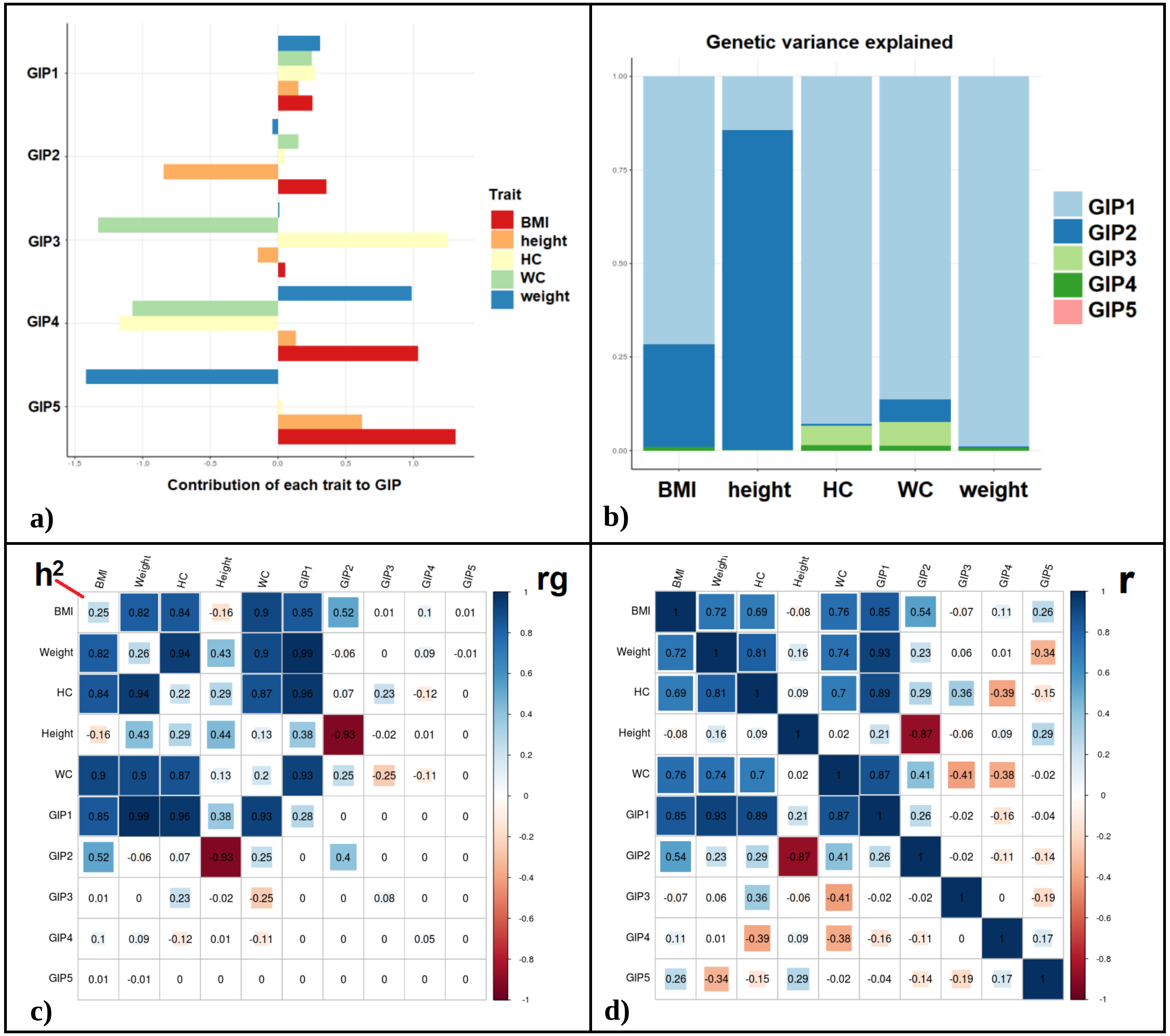

Figure SM4. Genetically independent phenotypes (GIP) for scenario (4).

a. Barplots depicting the contribution of the four original traits to each GIP. The bars represent orthogonal transformation coefficients. BMI, body mass index; HC, hip circumference; WC, waist circumference.

b. Genetic variance of the original traits explained by four GIPs.

c. Estimated matrix of genetic correlations (rg) between the original traits and GIPs. The diagonal elements represent estimates of SNP-based heritability (h^2) for each trait.

d. Matrix of phenotypic correlations (r) between the original phenotypes and GIPs.

Conclusion

As can be seen from Figure SM4b, GIP1 still explains the largest proportion of the genetic variance of BMI, weight, HC, and WC, but explains only a minor proportion of the genetic variance of height.

Also, in this case, heritability of GIP1 (0.28) is smaller than one of GIP2 (0.40).

(5) Results of analysis of four traits that are not strongly genetically dependent

We applied the GIP method to four traits that are not strongly genetically dependent (real data). The traits were selected as follows:

1) First, we selected the UK Biobank trait "varicose veins of lower extremities, ICD10 code I83" (VVs) which we have analyzed in our previous study ("Varicose veins of lower extremities: Insights from the first large-scale genetic study", https://doi.org/10.1371/journal.pgen.1008110). GWAS summary statistics for this trait were obtained from the Gene ATLAS database (http://geneatlas.roslin.ed.ac.uk/).

2) Second, we used our results of the genetic correlation analysis between VVs and 861 complex traits presented in the above-mentioned paper. We sorted these results by *P*-value for genetic correlations (from the largest to the smallest) and selected the top three traits with the lowest level of statistical significance of genetic correlation with VVs and for which we did not propose biological relations.

The list of selected traits:

- Heel bone mineral density (HBMD),
- Left-handed - Handedness, chirality/laterality (LH),
- Total lipids in large very low-density lipoproteins (TL_VLDL),
- Varicose veins of lower extremities (VVs).

The results of the GIP analysis are presented in Figure SM5. The results are very similar to the scenario (1) where the shared genetic background does not exist by definition.

N.B.!

Is should be noted that in each case of the application of the proposed GIP approach the results should be evaluated carefully by examining the plot of genetic variance of original traits explained by each GIPs and the plot of the estimated matrix of genetic correlations between the original traits and GIPs. This method does not provide solutions for all cases, and the decision on whether the shared genetic background can be approximated by GIP1 or not should be made based on the results obtained.

Also, it should be noted that this approach has mathematical restriction of the **minimal number of the studied traits, which should be not less than 3.**

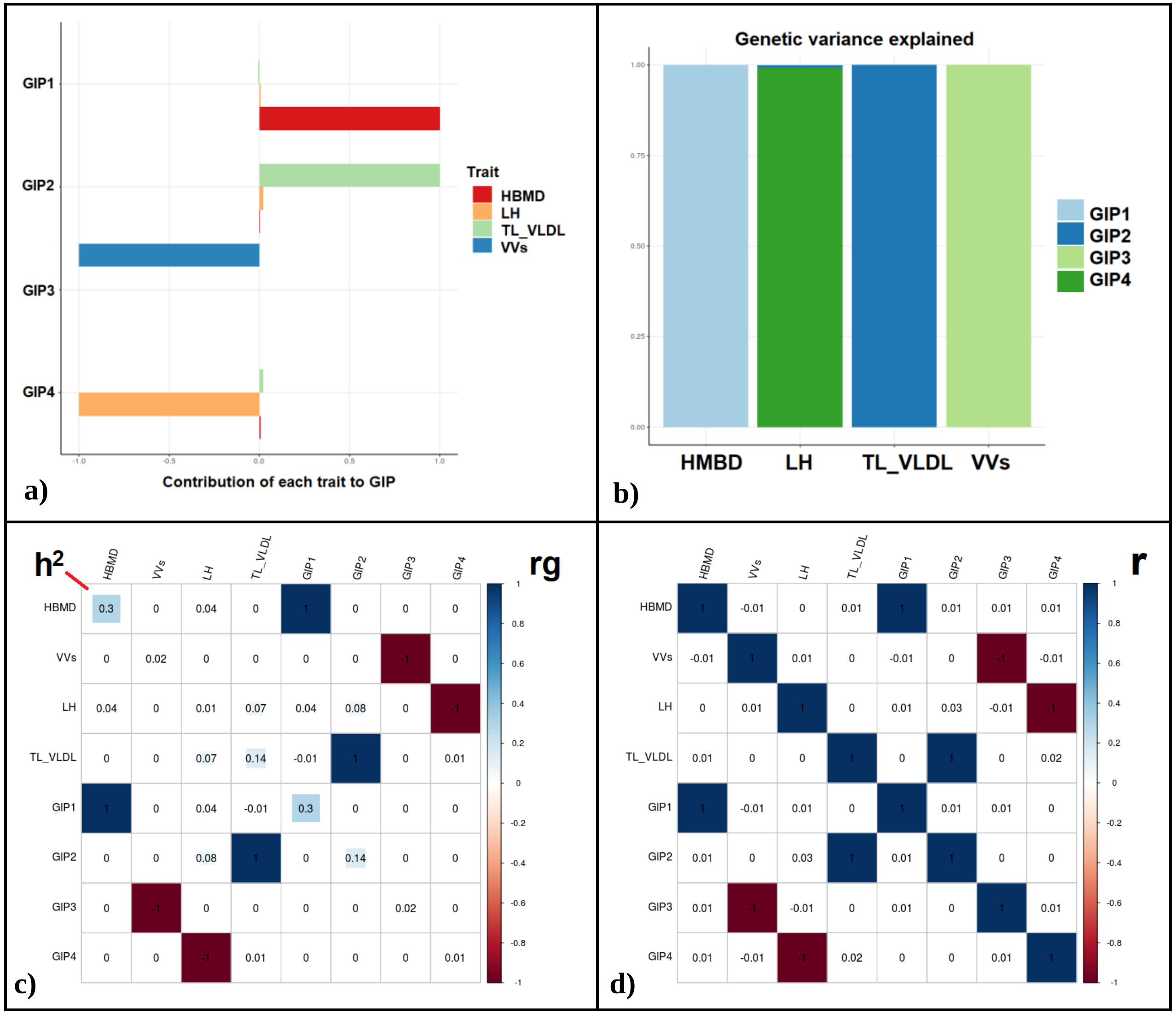

Figure SM5. Genetically independent phenotypes (GIP) for scenario (5).

a. Barplots depicting the contribution of the four original traits to each GIP. The bars represent orthogonal transformation coefficients.

HBMD, heel bone mineral density; LH, Left-handed – Handedness, chirality/laterality; TL_VLDL, total lipids in large very low-density lipoproteins; VVs, varicose veins of lower extremities.

b. Genetic variance of the original traits explained by four GIPs.

c. Estimated matrix of genetic correlations (rg) between the original traits and GIPs. The diagonal elements represent estimates of SNP-based heritability (h^2) for each trait.

d. Matrix of phenotypic correlations (r) between the original phenotypes and GIPs.

Testing for pleiotropy using SMR/HEIDI approach

SMR/HEIDI analysis was conducted as described by Zhu et al.[1] HEIDI statistics were calculated as $T_{HEIDI} = \sum_i^m z_{d(i)}^2$, where m is the number of SNPs selected for analysis, $z_{d(i)} = {d_i}/{SE_{(d_i)}}$ and $d_i = \beta_{SMR_i} - \beta_{SMR\ (lead\ SNP)}$.

SNP selection was performed as follows:

1) We defined a set of eligible markers within ±250 kb from the lead SNP in the primary GWAS, which had $\chi^2 > 10$ in the primary GWAS, and for which the results were reported in the secondary GWAS;
2) Made empty "target" and "rejected" SNP sets;
3) Selected SNP from the primary GWAS with the lowest *P*;
4) If this SNP had $r^2 > 0.9$ with any SNP in the target SNP set, we added it to the "rejected" set. LD matrix (r^2) was computed with PLINK 1.9 (https://www.cog-genomics.org/plink2) using 1000 Genomes data for 503 European individuals (http://www.internationalgenome.org/data/);
5) Otherwise, it was added to the "target" set;
6) Procedure was repeated from the step 3) until either eligible SNP set was exhausted, or the "target" set had 20 SNPs. If we could not select 3 or more SNPs, no test was performed.

When testing for pleiotropy with complex traits, we standardized all SNP effects (β) and standard errors (made the variances of the traits equal to 1): $\breve{\beta}_{Y_i} = \beta_{Y_i}/SD_{Y_i}$ and $\breve{SE}_{Y_i} = SE_{Y_i}/SD_{Y_i}$, where $\breve{\beta}_{Y_i}$ and $\breve{SE}_{Y_i}$ are standardized betas and standard errors for the trait Y_i; β_{Y_i} and SE_{Y_i} are original betas and standard errors for the trait Y_i; SD_{Y_i} is a square root of estimated variance of the trait Y_i. Analysis was conducted using Python 3.5 as the main programming language.

REFERENCES:

1. Zhu, Z. et al. Integration of summary data from GWAS and eQTL studies predicts complex trait gene targets. *Nat. Genet.* **48**, 481–487 (2016).

Supplementary Figure 1

Contribution of the four chronic musculoskeletal pain traits to each GIP. The violin plots depict the empirical distribution of the coefficients of orthogonal transformation. Points represent each coefficient, horizontal line represents median, white rectangle represents interquartile range, and width of each figure represents a smoothed density of points.

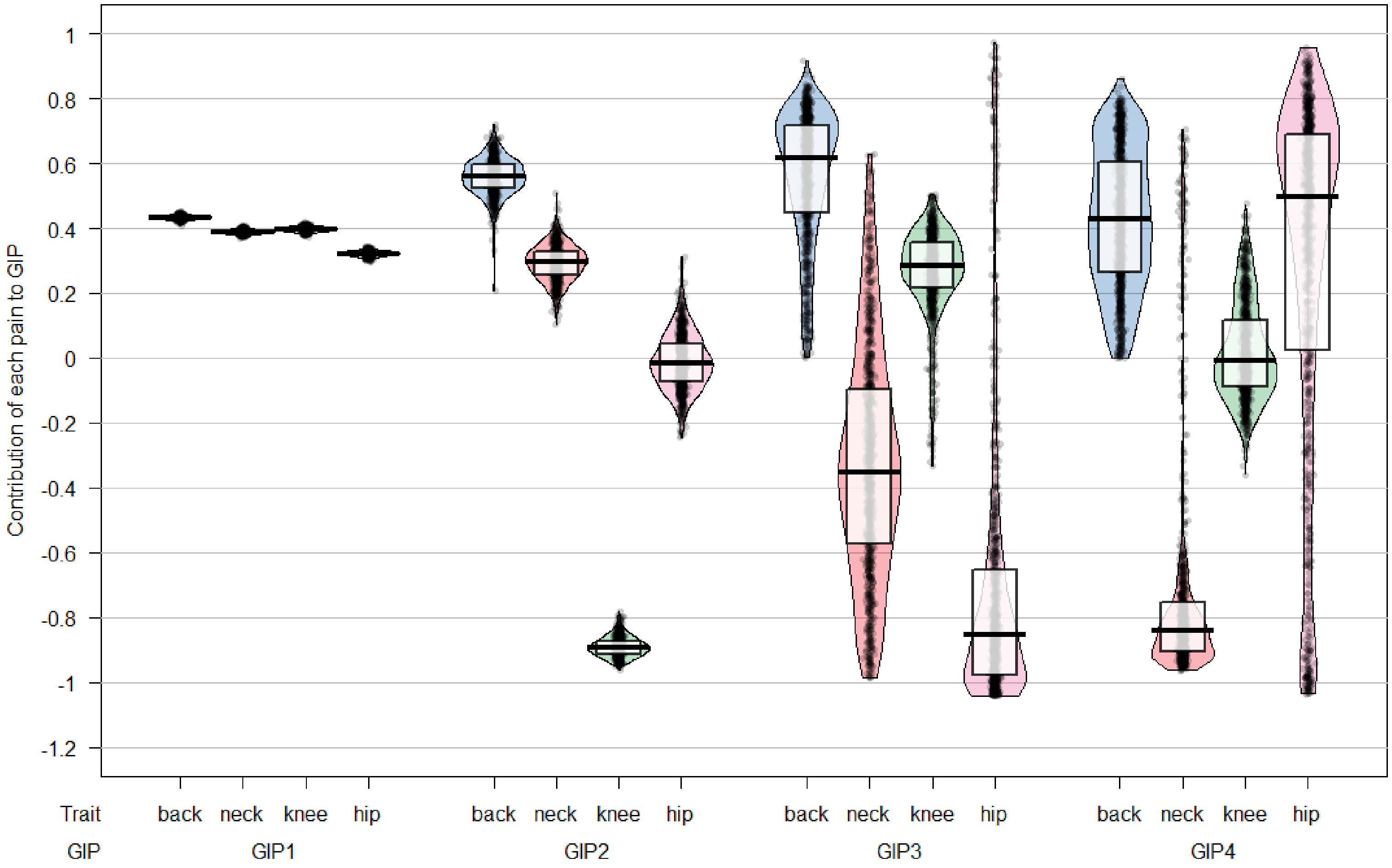

Supplementary Figure 2

Graphical summary of the discovery GWAS stage after the genomic control correction using LD Score regression intercept (for GIP2-GIP4).
Red line corresponds to the genome-wide significance threshold of P = 1.25e-08 (5.0e-08/4, where 4 is the number of GIPs). Only associations with P < 1.0e-02 are presented. Replicated loci are annotated.

GIP2

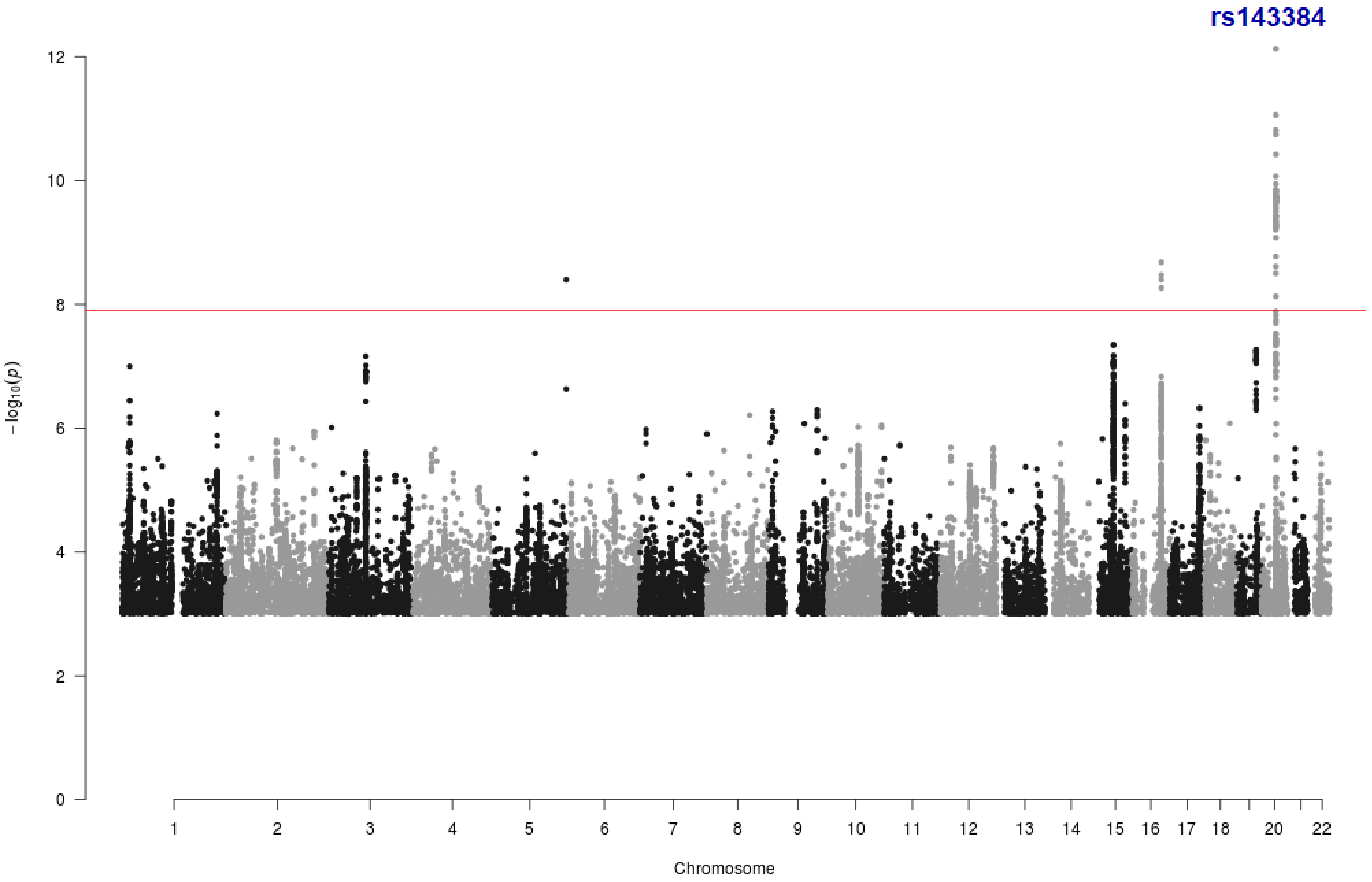

GIP3

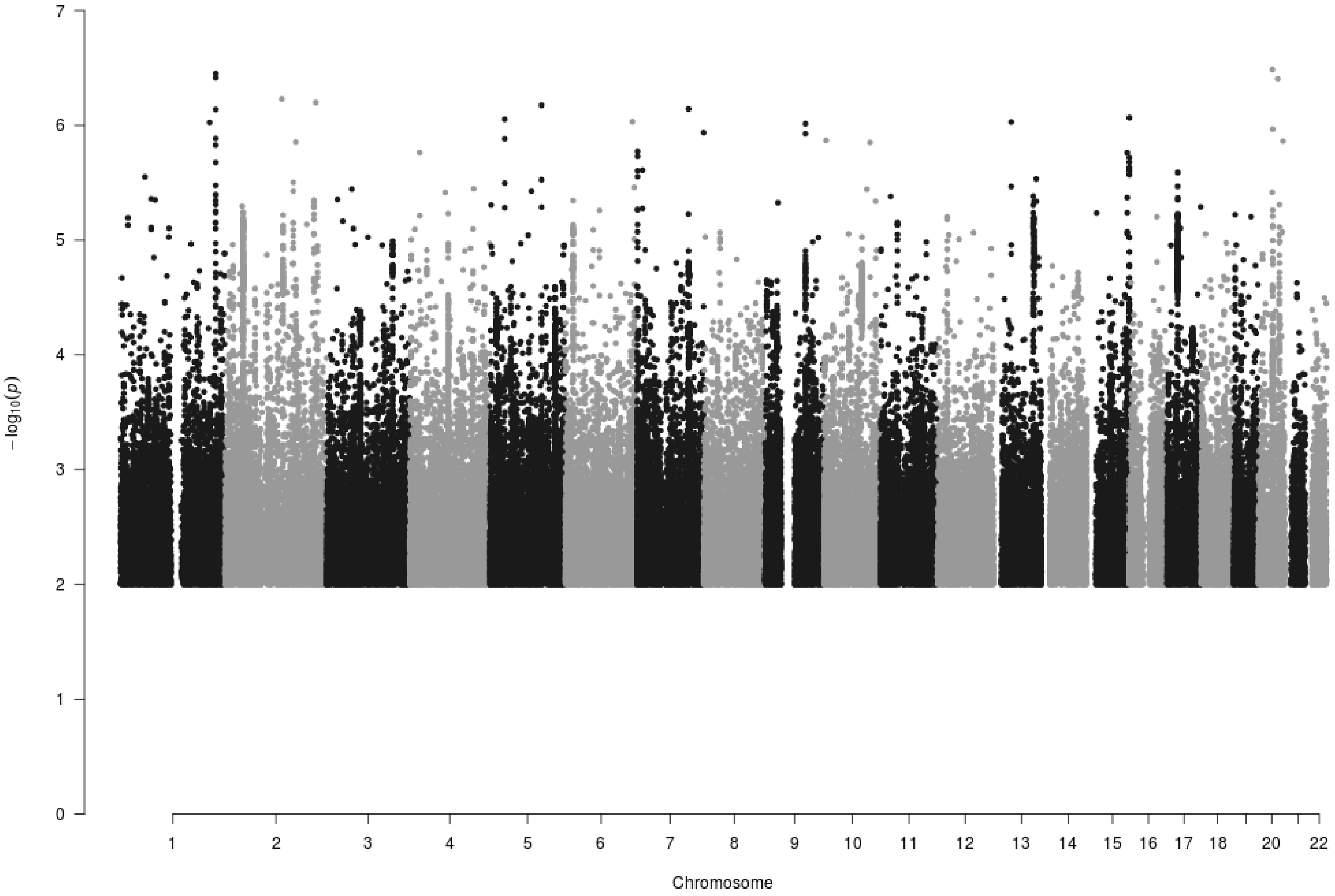

7
6
5
4
3
2
1
0
−log10(p)
1
2
3
4
5
6
7
8
9
10
11
12
13
14
15
16
17
18
20
22
Chromosome

GIP4

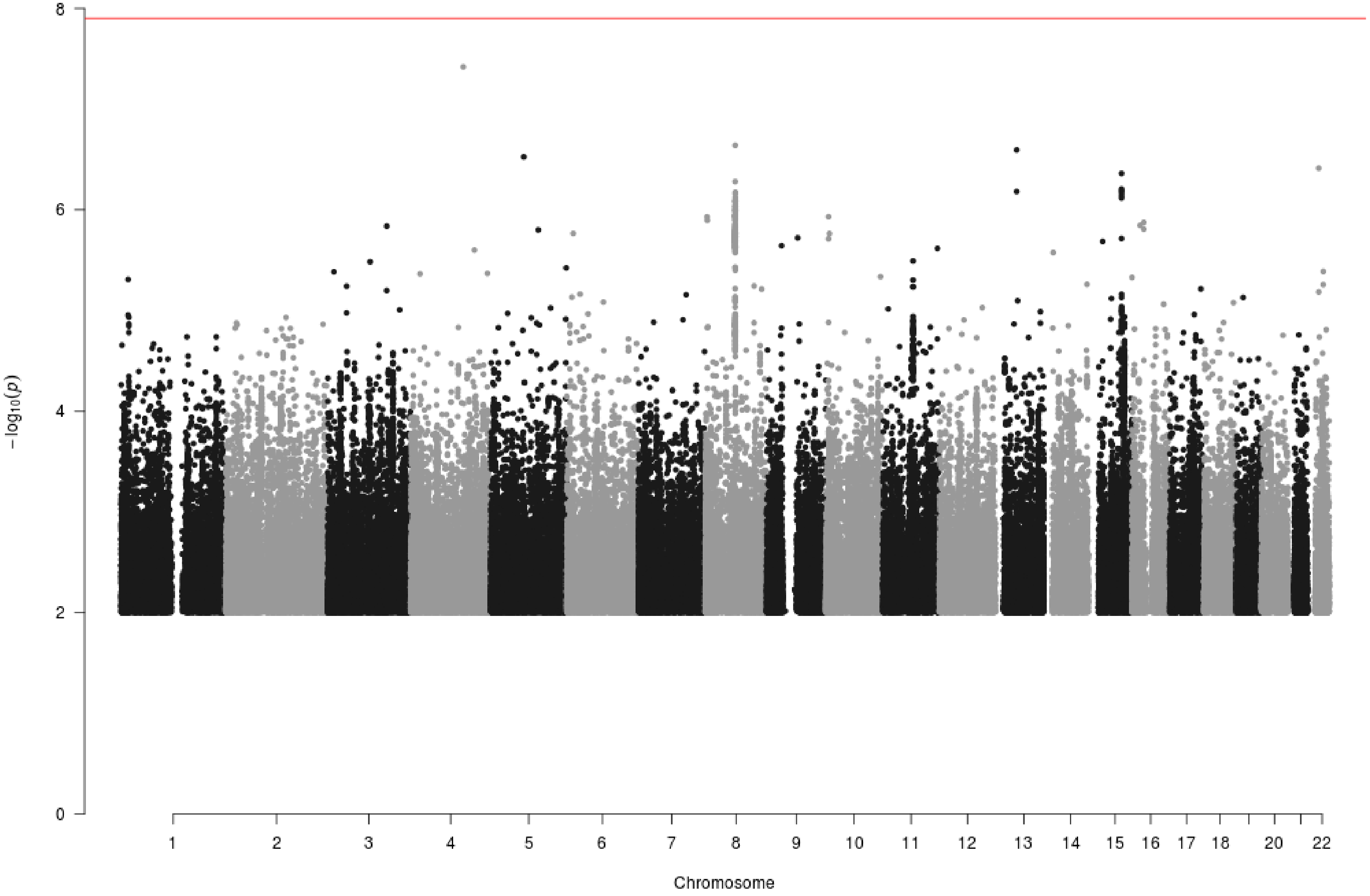

8
6
4
2
0
− log10(p)
1
2
3
4
5
6
7
8
9
10
11
12
13
14
15
16
17
18
20
22
Chromosome

Supplementary Figure 3

Quantile-quantile plots for observed vs. expected distribution of *P*-values for χ^2 statistics.

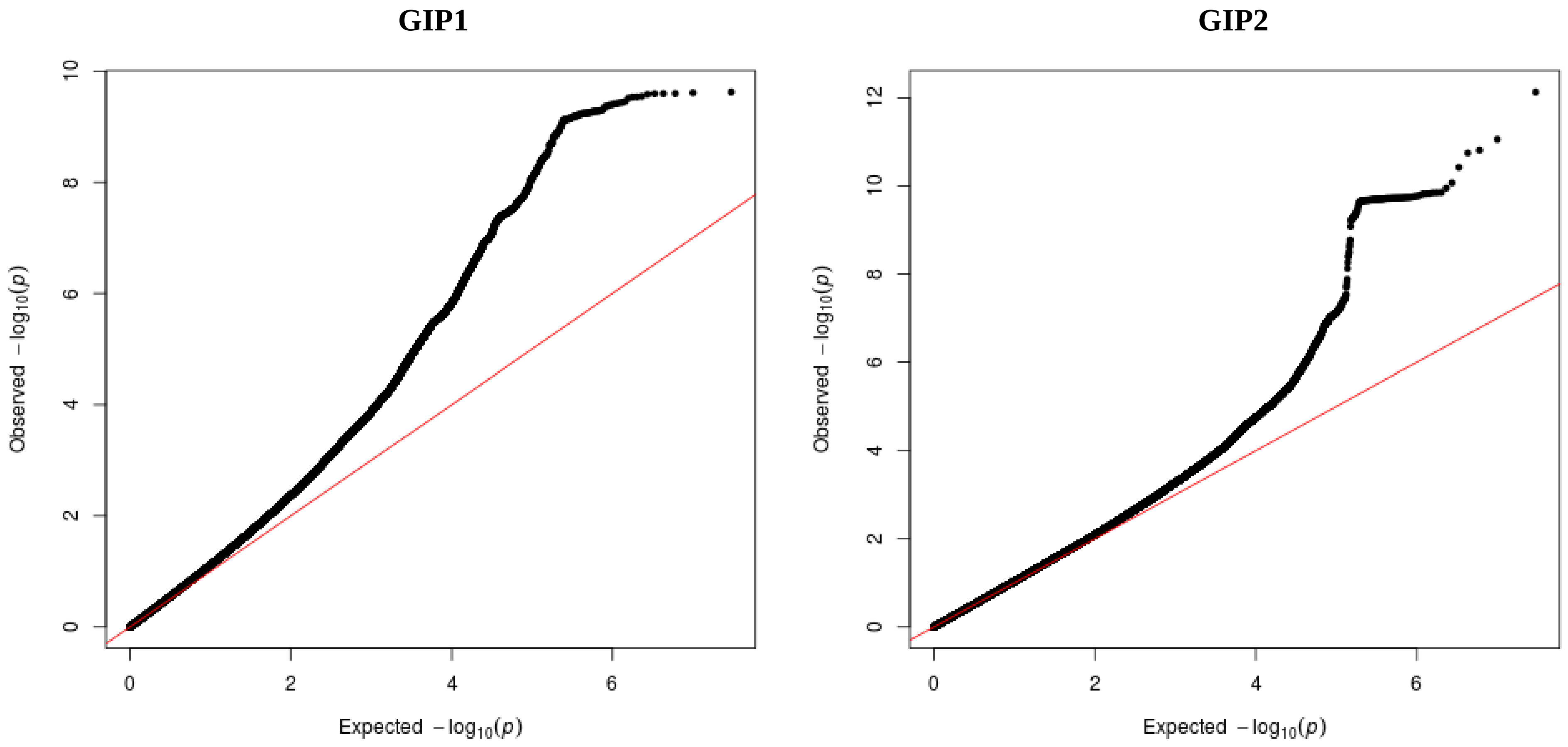

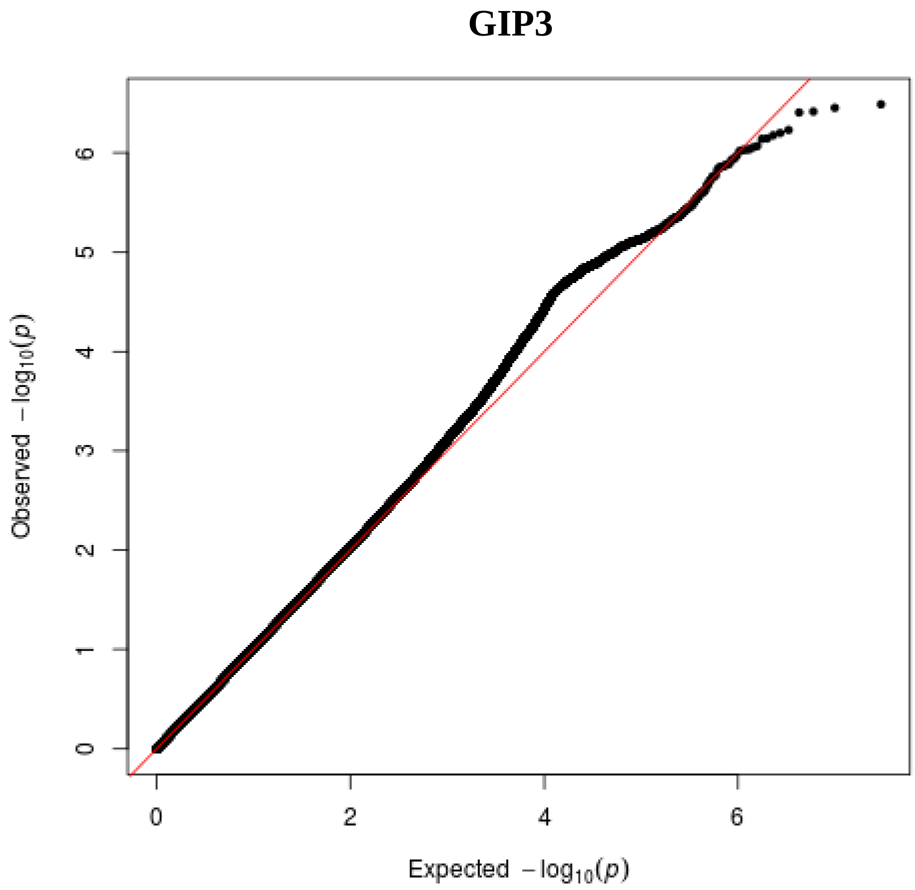
GIP3
Observed $-\log_{10}(p)$
Expected $-\log_{10}(p)$
0
1
2
3
4
5
6
0
2
4
6

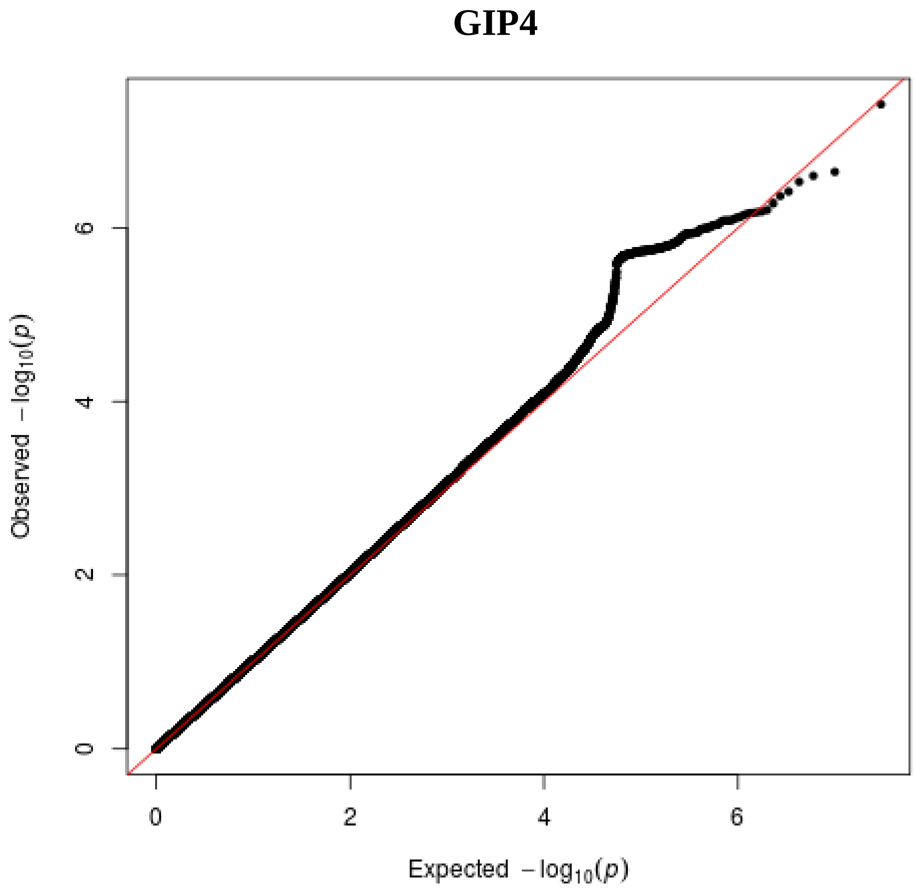
GIP4
Observed $-\log_{10}(p)$
Expected $-\log_{10}(p)$
0
2
4
6
0
2
4
6

Supplementary Figure 4

Regional association plots of $-\log_{10}(P)$ for SNPs located at the distance of ≤ 250 kb from lead SNPs. Color of circles indicates the strength of linkage disequilibrium with the lead SNP based on the squared correlation coefficient (r^2). Blue line indicates recombination rate (cM/Mb). Genes are indicated as blue bars under the plot.

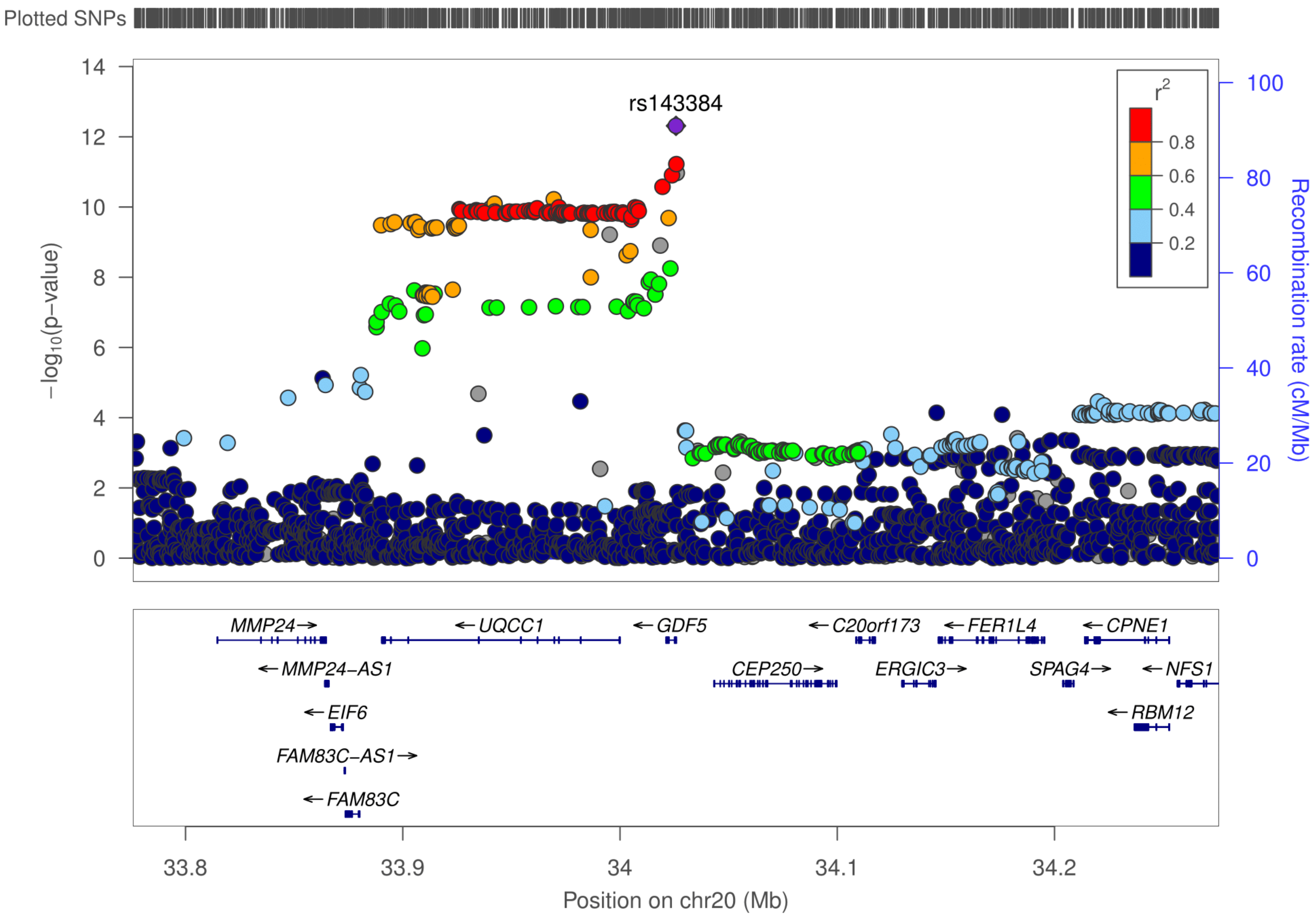

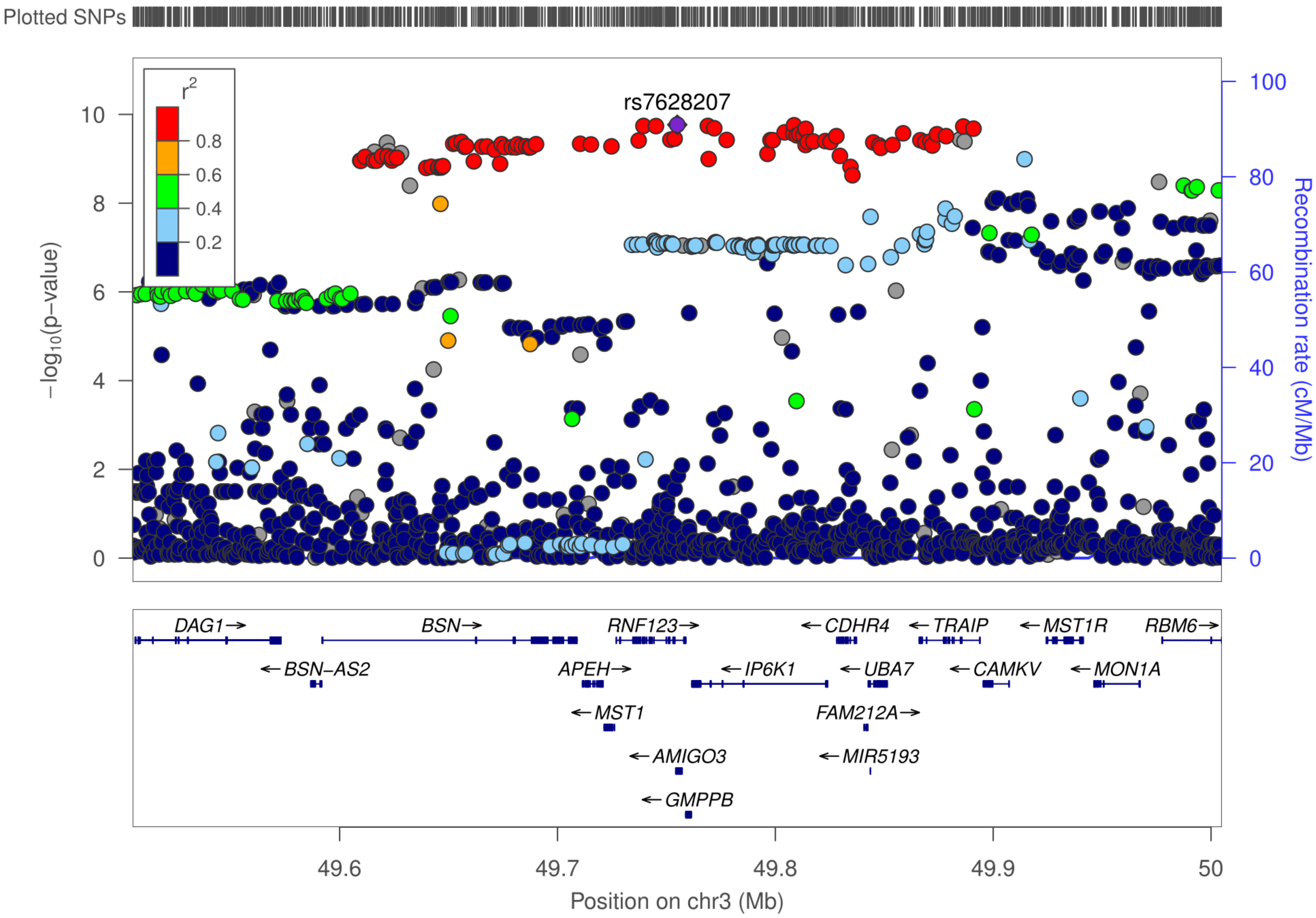

Plotted SNPs
rs7628207
r2
0.8
0.6
0.4
0.2
−log10(p−value)
Recombination rate (cM/Mb)
0
2
4
6
8
10
20
40
60
80
100
DAG1→
BSN→
RNF123→
←CDHR4
←TRAIP
←MST1R
RBM6→
←BSN−AS2
APEH→
←IP6K1
←UBA7
←CAMKV
←MON1A
←MST1
FAM212A→
←AMIGO3
←MIR5193
←GMPPB
49.6
49.7
49.8
49.9
50
Position on chr3 (Mb)

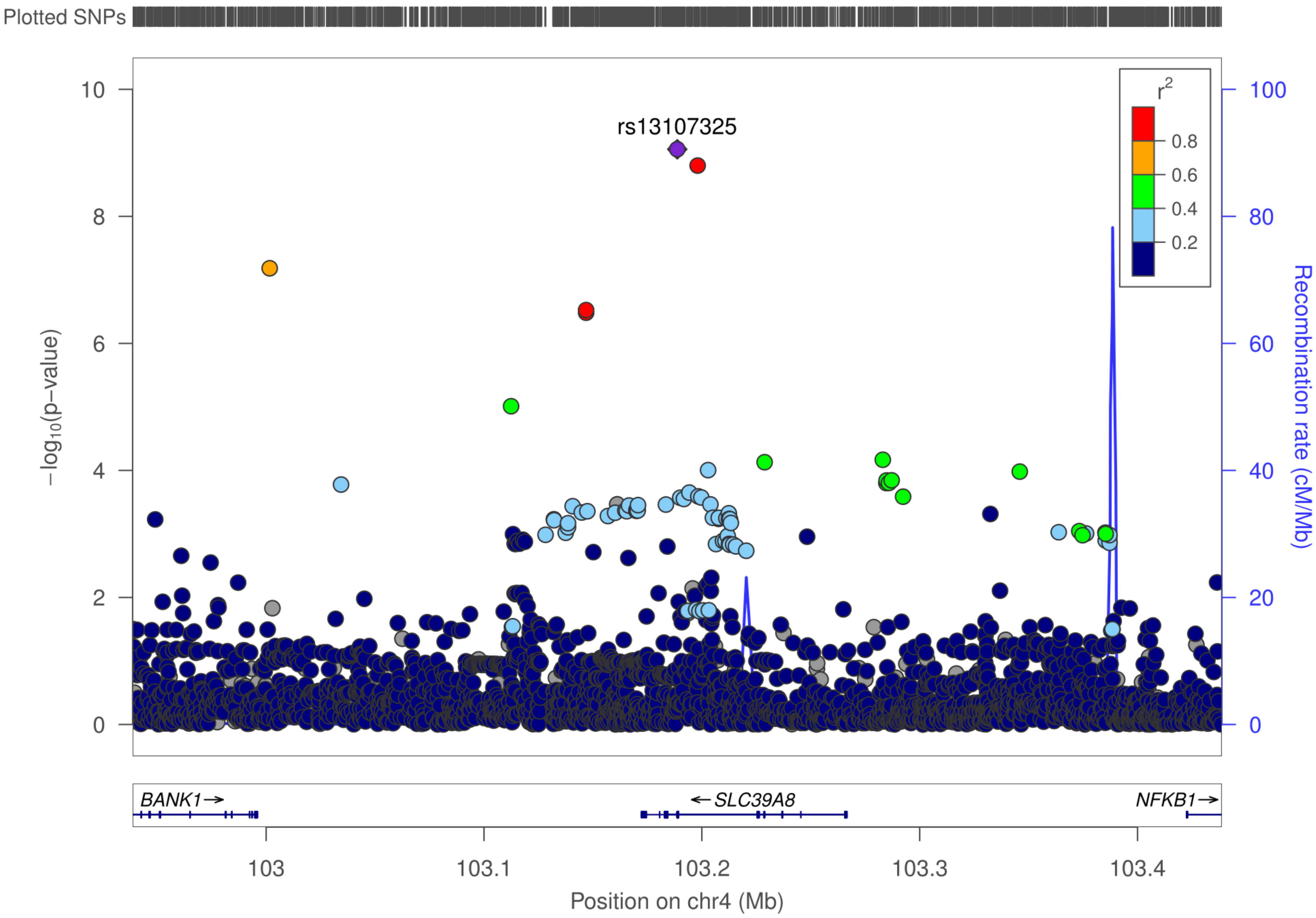
Plotted SNPs
rs13107325
r2
0.8
0.6
0.4
0.2
10
8
6
4
2
0
−log10(p−value)
100
80
60
40
20
0
Recombination rate (cM/Mb)
BANK1→
←SLC39A8
NFKB1→
103
103.1
103.2
103.3
103.4
Position on chr4 (Mb)

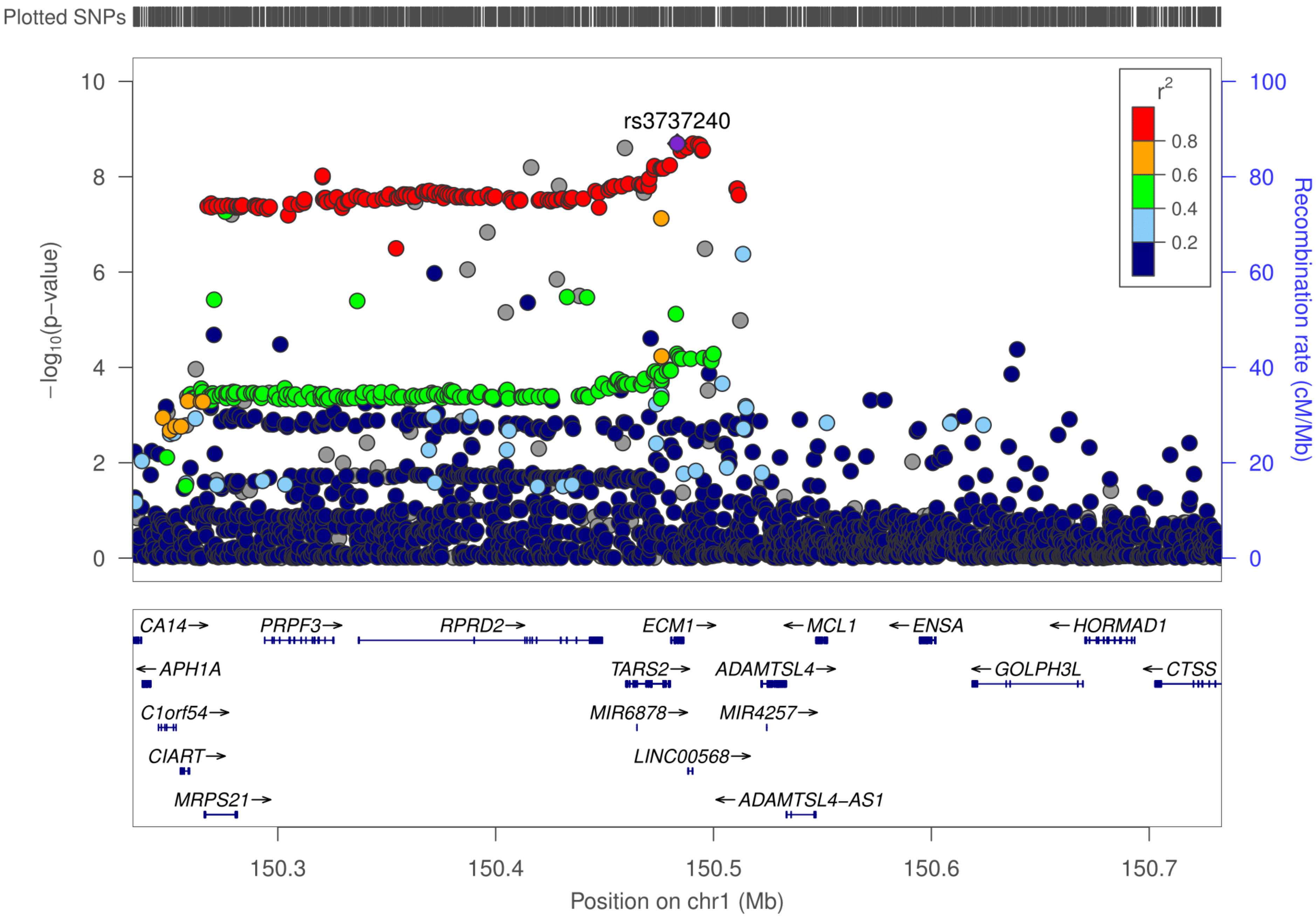
Plotted SNPs
rs3737240
r2
0.8
0.6
0.4
0.2
-log10(p-value)
Recombination rate (cM/Mb)
10
8
6
4
2
0
100
80
60
40
20
0
CA14→
PRPF3→
RPRD2→
ECM1→
←MCL1
←ENSA
←HORMAD1
←APH1A
TARS2→
ADAMTSL4→
←GOLPH3L
←CTSS
C1orf54→
MIR6878→
MIR4257→
CIART→
LINC00568→
MRPS21→
←ADAMTSL4-AS1
150.3
150.4
150.5
150.6
150.7
Position on chr1 (Mb)

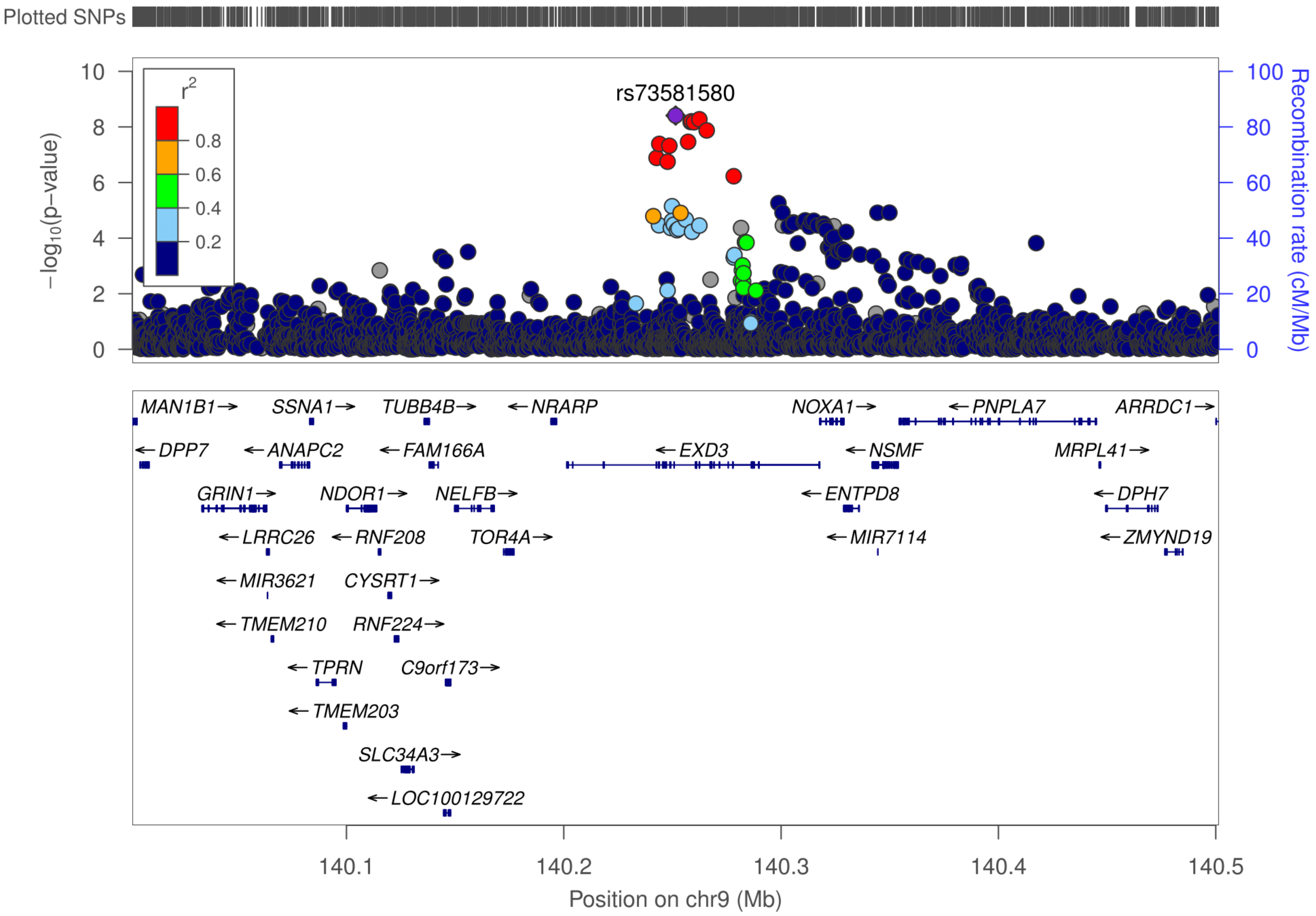
Plotted SNPs
rs73581580
r2
0.8
0.6
0.4
0.2
−log10(p−value)
Recombination rate (cM/Mb)
10
8
6
4
2
0
100
80
60
40
20
0
MAN1B1
SSNA1
TUBB4B
NRARP
NOXA1
PNPLA7
ARRDC1
DPP7
ANAPC2
FAM166A
EXD3
NSMF
MRPL41
GRIN1
NDOR1
NELFB
ENTPD8
DPH7
LRRC26
RNF208
TOR4A
MIR7114
ZMYND19
MIR3621
CYSRT1
TMEM210
RNF224
TPRN
C9orf173
TMEM203
SLC34A3
LOC100129722
140.1
140.2
140.3
140.4
140.5
Position on chr9 (Mb)

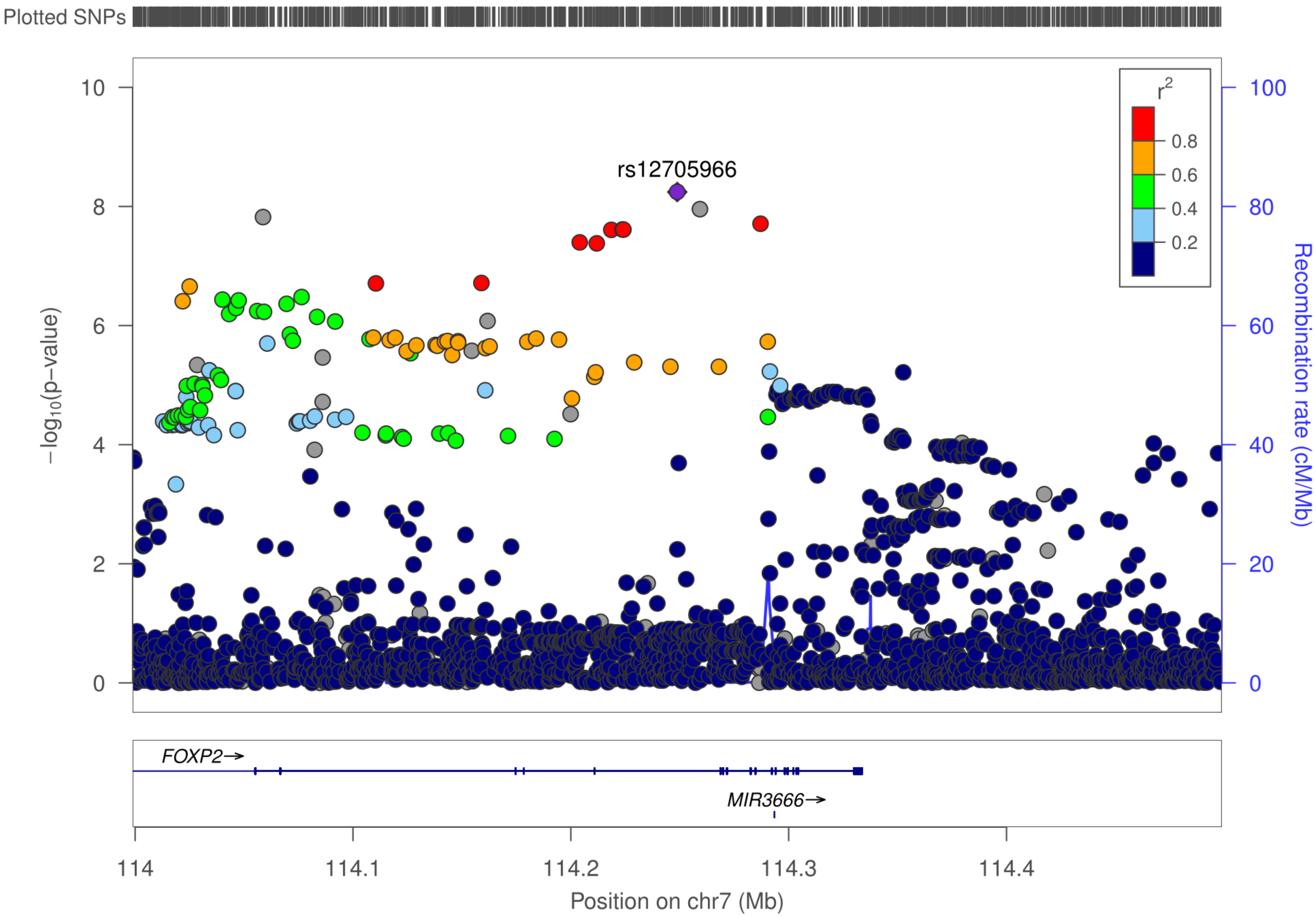
Plotted SNPs
rs12705966
r²
0.8
0.6
0.4
0.2
−log10(p−value)
Recombination rate (cM/Mb)
10
8
6
4
2
0
100
80
60
40
20
0
FOXP2→
MIR3666→
114
114.1
114.2
114.3
114.4
Position on chr7 (Mb)

Supplementary Figure 5

Results of tissue enrichment analysis for GIP1 performed using the FUMA platform (doi:10.1038/s41467-017-01261-5).

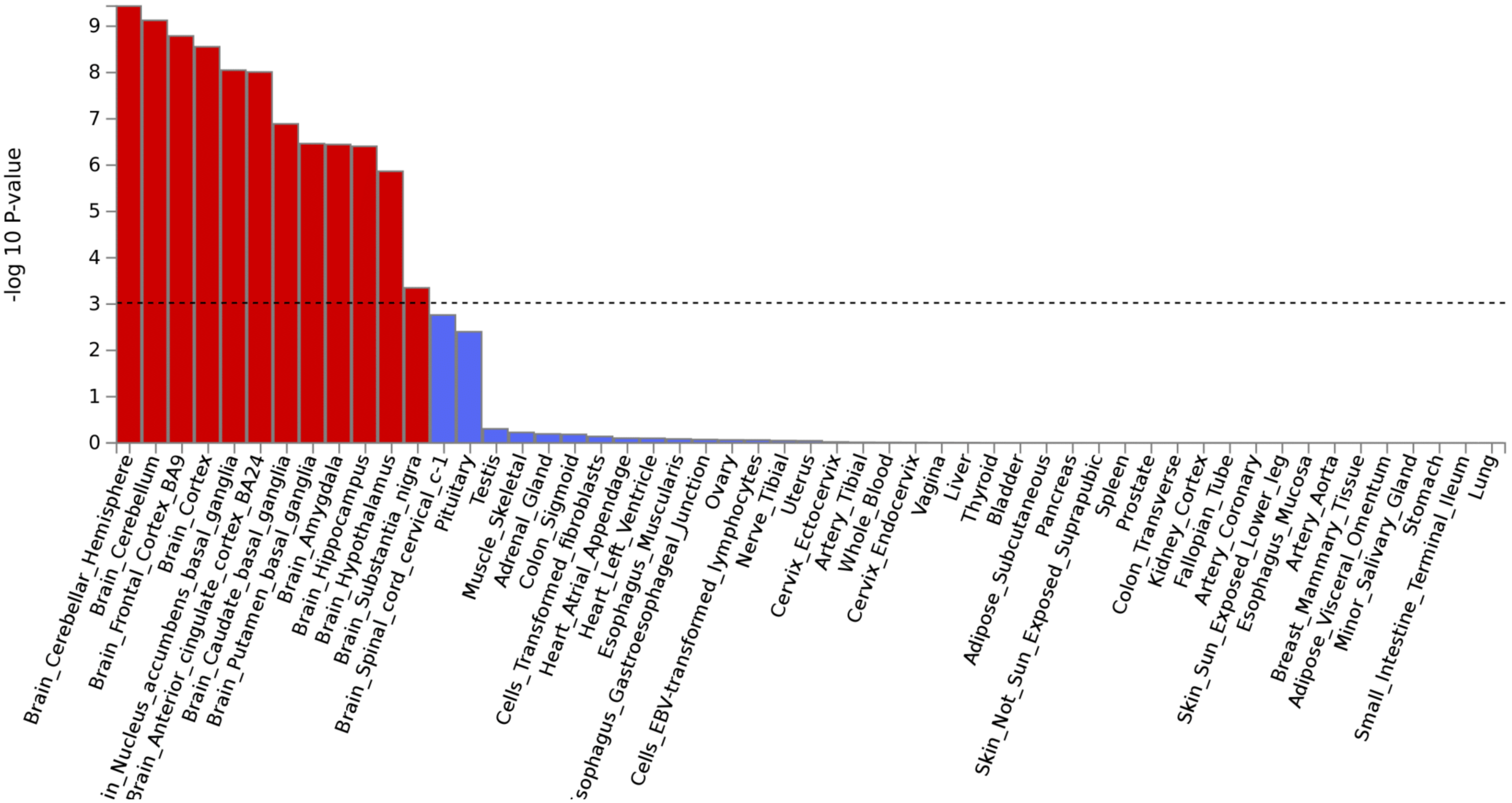

Supplementary Figure 6

Matrix of genetic correlations between GIPs, chronic musculoskeletal pain traits and hospital-diagnosed osteoarthritis (the UK Biobank trait for which GWAS summary statistics were downloaded from the Michigan PheWeb database, http://pheweb.sph.umich.edu/SAIGE-UKB/pheno/740). Color depicts the sign and absolute value of the genetic correlation coefficients (rg).

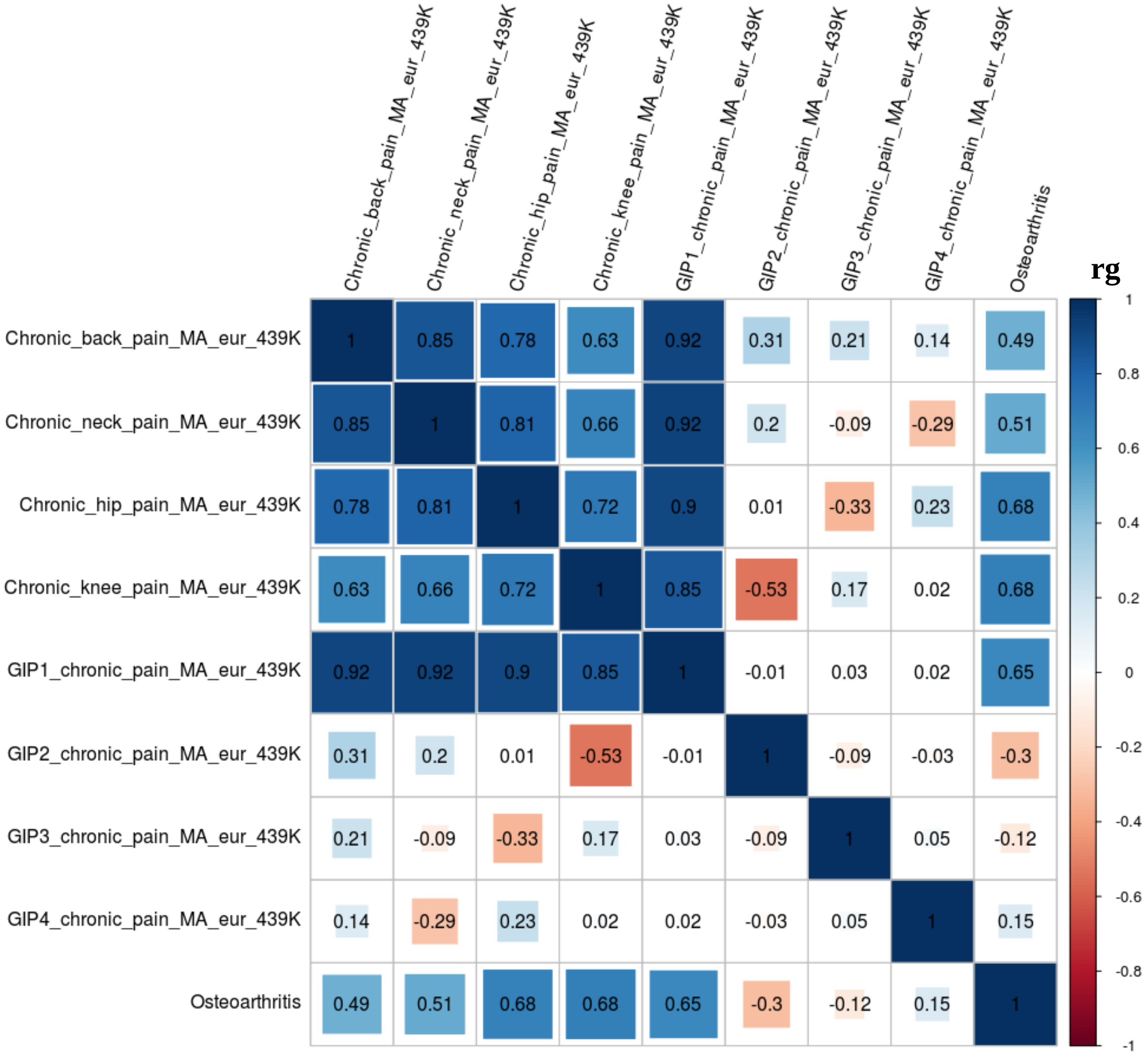

Supplement: Supplementary file 1 — Supplementary Information [file 42003_2020_1051_MOESM1_ESM.pdf]
