# Supplementary material for: Analysis of genetically independent phenotypes identifies shared genetic factors associated with chronic musculoskeletal pain conditions
Source: Commun Biol. 2020 Jun 25;3:329. doi: 10.1038/s42003-020-1051-9 (PMC7316754; doi:10.1038/s42003-020-1051-9)
Supplement: Supplementary file 5 — Supplementary Data 4 [file 42003_2020_1051_MOESM5_ESM.doc]

**Supplementary Table 4.** Literature data on well-studied SNPs associated with GIP1 and GIP2.

| **SNP** | **GIP, allele^*^** | **Description** | **Functional effect** | **Association with complex traits** |
| --- | --- | --- | --- | --- |
| **rs13107325** | GIP1, allele T | Missense SNP in the *SLC39A8* gene (encoding a divalent cation transporter), C>T nucleotide substitution causes Ala391Thr amino acid change.   - This SNP is a triallelic SNP (C>A, T).   A very rare allele A leads to Ala391Ser amino acid change.  Note: rs13107325 is one of the most pleiotropic variants in the human genome | Unknown | **Allele rs13107325 T:**  Crohn's disease (↑ risk) and human gut microbiome composition^1^  Schizophrenia (↑ risk)^2^  Severe adolescent idiopathic scoliosis (↑ risk)^3^  Osteoarthritis (↑ risk)^4^  Decreased height^3^  Increased body mass index^3,5^  Greater spinal curvature^3^  Lower plasma manganese^3,6^  Decreased capacity for cell Mn^2+^ uptake^3^  Increased plasma levels of N-terminal pro-B-type natriuretic peptide in patients with acute coronary syndromes^7^  Decreased HDL cholesterol^8, 9^  Reduced blood pressure phenotypes and ↓ risk of hypertension^10,11^ |
| **rs3737240** | GIP1, allele C | Missense SNP in the *ECM1* (extracellular matrix protein 1) gene, C>T nucleotide substitution causes Thr130Met amino acid change in the collagen IV binding domain of the ECM1 protein.  Rs3737240 is tightly linked with **rs13294** (allele rs3737240 T is positively correlated with allele rs13294 A, D’ = 1.00, r^2^ = 0.97 in European ancestry populations).  Rs13294 is also a missense *ECM1* variant, G>A nucleotide substitution causes Gly290Ser amino acid change.   - Rs13294 is a triallelic SNP (G>A, T).   A very rare allele T leads to Gly290Cys amino acid change. | Unknown | **Allele rs3737240 T or rs13294 A:**  Ulcerative colitis (↑ risk)^12-14^  **Allele rs3737240 C:**  is positively correlated with allele rs12040949 C (r^2^ = 0.94 in European ancestry populations). Allele rs12040949 C is associated with hip osteoarthritis (↑ risk)^4^ |
| **rs143384** | GIP2, allele C | Rs143384 (T>C) is located in the 5’-untranslated region of the *GDF5* (growth differentiation factor 5) gene.  Rs143384 is in high LD with its neighboring SNP **rs143383^**^** (T>C, allele rs143384 T is positively correlated with allele rs143383 T,  D’ = 0.99, r^2^ = 0.82 in European ancestry populations). | Rs143383 exerts joint-wide effect on the *GDF5* gene expression (allele rs143383 T is associated with decreased expression level)^15^.  The study using luciferase reporter assays demonstrated that rs143384 can modulate the effect of rs143383 *in vitro*^15^. | **Allele rs143383 T:**  Osteoarthritis (↑ risk)^4,15-19^  Congenital dislocation of the hip (↑ risk)^20,21^  Lumbar disc degeneration (↑ risk)^22^ |

*Genetically independent phenotype (GIP) associated with SNP; allele positively associated with this GIP.

**In our study, association of rs143383 with GIP2 had the same magnitude of effect as that of rs143384, and also passed the study-level statistical significance threshold (discovery cohort: *P* = 8.53e-12 after correction for residual inflation). It should be noted that both variant alleles (rs143384 T and rs143383 T) were inversely associated with GIP2, consistent with the negative coefficient of knee pain phenotype observed for GIP2 (Figure 2a; Supplementary Figure 1).

REFERENCES:

1. Li, D. *et al.* A pleiotropic missense variant in SLC39A8 is associated with Crohn’s disease and human gut microbiome composition. *Gastroenterology* **151**, 724–732 (2016).

2. Carrera, N. *et al*. Association study of nonsynonymous single nucleotide polymorphisms in schizophrenia. *Biol. Psychiatry* **71**, 169–177 (2012).

3. Haller, G. *et al.* A missense variant in SLC39A8 is associated with severe idiopathic scoliosis. *Nat. Commun.* **9**, 4171; 10.1038/s41467-018-06705-0 (2018).

4. Tachmazidou, I. *et al.* Identification of new therapeutic targets for osteoarthritis through genome-wide analyses of UK Biobank data. *Nat. Genet.* **51**, 230–236 (2019).

5. Speliotes, E. K. *et al.* Association analyses of 249,796 individuals reveal 18 new loci associated with body mass index. *Nat. Genet.* **42**, 937–948 (2010).

6. Ng, E. *et al.* Genome-wide association study of toxic metals and trace elements reveals novel associations. *Hum. Mol. Genet.* **24**, 4739–4745 (2015).

7. Johansson, Å. *et al.* Genome-wide association and Mendelian randomization study of NT-proBNP in patients with acute coronary syndrome. *Hum. Mol. Genet.* **25**, 1447–1456 (2016).

8. Teslovich, T. M. *et al.* Biological, clinical and population relevance of 95 loci for blood lipids. *Nature* **466**, 707–713 (2010).

9. Willer, C. J. *et al.* Discovery and refinement of loci associated with lipid levels. *Nat. Genet.* **45**, 1274–1283 (2013).

10. Ehret, G. B. *et al.* Genetic variants in novel pathways influence blood pressure and cardiovascular disease risk. *Nature* **478**, 103–109 (2011).

11. Wain, L. V *et al.* Genome-wide association study identifies six new loci influencing pulse pressure and mean arterial pressure. *Nat. Genet.* **43**, 1005–1011 (2011).

12. Fisher, S. A. *et al.* Genetic determinants of ulcerative colitis include the ECM1 locus and five loci implicated in Crohn’s disease. *Nat. Genet.* **40**, 710–712 (2008).

13. Festen, E. A. *et al*. Genetic analysis in a Dutch study sample identifies more ulcerative colitis susceptibility loci and shows their additive role in disease risk. *Am. J. Gastroenterol.* **105**, 395–402 (2010).

14. Adali, G. *et al.* Extracellular matrix protein 1 gene rs3737240 single nucleotide polymorphism is associated with ulcerative colitis in Turkish patients. *Turkish J. Gastroenterol.* **28**, 254–259 (2017).

15. Egli, R. J. *et al.* Functional analysis of the osteoarthritis susceptibility-associated *GDF5* regulatory polymorphism. *Arthritis Rheum.* **60**, 2055–2064 (2009).

16. Miyamoto, Y. *et al.* A functional polymorphism in the 5′ UTR of GDF5 is associated with susceptibility to osteoarthritis. *Nat. Genet.* **39**, 529–533 (2007).

17. Southam, L. *et al.* An SNP in the 5′-UTR of GDF5 is associated with osteoarthritis susceptibility in Europeans and with in vivo differences in allelic expression in articular cartilage. *Hum. Mol. Genet.* **16**, 2226–2232 (2007).

18. Chapman, K. *et al.* A meta-analysis of European and Asian cohorts reveals a global role of a functional SNP in the 5’ UTR of GDF5 with osteoarthritis susceptibility. *Hum. Mol. Genet.* **17**, 1497–1504 (2008).

19. Evangelou, E. *et al.* Large-scale analysis of association between *GDF5* and *FRZB* variants and osteoarthritis of the hip, knee, and hand. *Arthritis Rheum.* **60**, 1710–1721 (2009).

20. Dai, J. *et al.* Association of a single nucleotide polymorphism in growth differentiate factor 5 with congenital dysplasia of the hip: a case-control study. *Arthritis Res. Ther.* **10**, R126; 10.1186/ar2540 (2008).:

21. Rouault, K. *et al.* Evidence of association between GDF5 polymorphisms and congenital dislocation of the hip in a Caucasian population. *Osteoarthr. Cartil.* **18**, 1144–1149 (2010).

22. Williams, F. M. K. *et al.* GDF5 single-nucleotide polymorphism rs143383 is associated with lumbar disc degeneration in Northern European women. *Arthritis Rheum.* **63**, 708–12 (2011).
