# Supplementary material for: Analysis of genetically independent phenotypes identifies shared genetic factors associated with chronic musculoskeletal pain conditions
Source: Commun Biol. 2020 Jun 25;3:329. doi: 10.1038/s42003-020-1051-9 (PMC7316754; doi:10.1038/s42003-020-1051-9)
Supplement: Supplementary file 6 — Supplementary Data 5 [file 42003_2020_1051_MOESM6_ESM.docx]

**Supplementary Table 5.** Gene prioritization based on a literature review. Full protein names are indicated in bold italics.

| **Lead SNP** | **Candidate gene** official name (alternative name) | **OMIM^*^ code** | **Nearest gene?** | **Functional effects of the encoded proteins** |
| --- | --- | --- | --- | --- |
| rs143384 | *GDF5*  (*CDMP1*) | 601146 | **YES**; utr variant 5 prime | ***Growth differentiation factor 5***  Regulates the development of numerous tissue and cell types, including cartilage, joints, brown fat, teeth, and the growth of neuronal axons and dendrites. GDF5 plays an important role in knee morphology^1^. *GDF5* gene has multiple different control sequences that show striking specificity for joints in the head, vertebral column, shoulder, elbow, wrist, hip, knee, and digits^2^.  Mutations in the *GDF5* genes underlie rare skeletal disorders including Chondrodysplasia Grebe type^3^, Acromesomelic chondrodysplasia, Hunter-Thompson type^4^, and others. |
|  | *MMP24* | 604871 | NO; 160 kb from lead SNP | ***Matrix metallopeptidase 24***  MMP-24 is involved in the proteolytic degradation of extracellular matrix in normal physiological processes as well as in disease such as arthritis and metastasis.  Evidence from mice studies:   - MMP-24 (MT5-MMP) is expressed in differentiated neurons and regulates axonal growth^5^ - MMP-24 is an essential mediator of peripheral thermal nociception and inflammatory hyperalgesia^6^ - Expression of MMP-24 immediately and gradually increased in the spinal cord following peripheral partial sciatic nerve ligation^7^ - MMP-24 is essential for the development of mechanical allodynia and plays an important role in neuronal plasticity^8^ |
| rs7628207 | *AMIGO3* | 615691 | **YES**; intron variant | ***Adhesion molecule with Ig like domain 3***  AMIGO3 participates in the NgR1-p75/TROY receptor complex (substitutes for LINGO-1 protein). NgR1-p75/TROY-AMIGO3 mediates myelin-induced inhibition of axon growth in the acute phase of adult central nervous system injury^9^.  Suppression of AMIGO3 disinhibits the growth of axotomized dorsal root ganglion neurons and enables neurotrophin-3 to stimulate the regeneration of spinal cord dorsal column axons^10^. |
|  | *BSN* | 604020 | NO; 46 kb from lead SNP | ***Bassoon presynaptic cytomatrix protein***  BSN is thought to be a scaffolding protein involved in organizing the presynaptic cytoskeleton. *BSN* gene is expressed primarily in neurons in the brain.  In mutant mice, loss of BSN causes a reduction in normal synaptic transmission^11^. |

| rs13107325 | *SLC39A8*  *(ZIP8)* | 608732 | **YES**; missense | ***Solute carrier family 39 member 8***  Transmembrane protein that acts as a transporter of several divalent cations, including manganese (Mn^2+^), zinc (Zn^2+^), cadmium (Cd^2+^), and iron (Fe^2+^) across the plasma membrane.  SLC39A8 was proposed to be involved in osteoarthritis-related cartilage degradation through modulation of Zn^2+^ concentration, which is required for catalytic activity of MMP-13^12^. SLC39A8 (ZIP8) was upregulated in osteoarthritis cartilage of humans and mice. Ectopic expression of SLC39A8 in mouse cartilage tissue caused osteoarthritis cartilage destruction, whereas Zip8 knockout suppressed surgically induced osteoarthritis pathogenesis^13^. Down-regulating of SLC39A8 was shown to reduce cartilage destruction, that could be gained through targeting SLC39A8 by microRNA-488^12^.  In a zebrafish model, disruptive *slc39a8* gene mutation caused spinal abnormalities including thoracic spinal curvature and caudal vertebral fusions, impaired growth, and decreased motor activity^14^. |
| --- | --- | --- | --- | --- |
| rs3737240 | *MIR6878* | N/A | NO; 18 kb from lead SNP | MicroRNA 6878 was found to be differentially expressed in synovial fluid of male patients with osteoarthritis^15^. |
|  | *ECM1* | 602201 | **YES**; missense | ***Extracellular matrix protein 1***  ECM1 is involved in endochondral bone formation and cartilage development (is a negative regulator of bone mineralization and chondrogenesis)^16-18^, promotes angiogenesis^19^, inhibits MMP9 proteolytic activity^20^, interacts with many extracellular and structural proteins and contributes to the maintenance of skin integrity^21^, is a potential activator of NF-kB signaling^22^. |
|  | *CTSS* | 116845 | NO; 219 kb from lead SNP | ***Cathepsin S***  Lysosomal cysteine proteinase that can remodel extracellular matrix in various tissues.  Cathepsin S is critical for the maintenance of neuropathic pain and spinal microglia activation^23-26^.  Cathepsin S is responsible for Th1 cell-dependent transition of nerve injury-induced acute pain to a chronic pain state^27^. |
| rs73581580 | *MIR7114* | N/A | NO; 93 kb from lead SNP | MicroRNA 7114 was found to be differentially expressed in T-cells from patients with ankylosing spondylitis^28^. |
|  | *NSMF (NELF)* | 608137 | NO; 91 kb from lead SNP | ***NMDA receptor synaptonuclear signaling and neuronal migration factor***  NSMF was proposed to be involved in guidance of olfactory axon projections and migration of luteinizing hormone-releasing hormone neurons^29^.  Nuclear import of NSMF (Jacob protein) is important for dendrite development in the hippocampus^30^. |

|  | *NOXA1* | 611255 | NO; 66 kb from lead SNP | ***NADPH oxidase activator 1***  NOXA1 (member of the Nox family) activates NADPH oxidases, which catalyze a reaction generating reactive oxygen species.  Animal studies suggest that Nox enzymes contribute to signaling pathways involved in chronic inflammatory and/or neuropathic pain states^31^. |
| --- | --- | --- | --- | --- |
|  | *GRIN1* | 138249 | NO; 218 kb from lead SNP | ***Glutamate ionotropic receptor NMDA type subunit 1***  Critical subunit of N-methyl-D-aspartate receptors. These subunits play an important role in the plasticity of synapses, which is believed to underlie memory and learning^32^.  A target for analgesic drugs^33^. |
| rs12705966 | *FOXP2* | 605317 | **YES**; intron variant | ***Forkhead box P2***  FOXP2 is required for proper development of speech and language regions of the brain during embryogenesis. Mutations in FOXP2 cause developmental speech and language disorders in humans^34,35^. |

^*^Online Mendelian Inheritance in Man database (<https://www.omim.org/>)

REFERENCES:

1. Pregizer, S. K. et al. Impact of broad regulatory regions on Gdf5 expression and function in knee development and susceptibility to osteoarthritis. *Ann. Rheum. Dis.* **77**, 450–450 (2018).

2. Chen, H. *et al*. Heads, Shoulders, elbows, knees, and toes: Modular Gdf5 enhancers control different joints in the vertebrate skeleton. *PLOS Genet.* **12**, e1006454; 10.1371/journal.pgen.1006454 (2016).

3. Thomas, J. T. *et al.* Disruption of human limb morphogenesis by a dominant negative mutation in CDMP1. *Nat. Genet.* **17**, 58–64 (1997).

4. Thomas, J. T. *et al.* A human chondrodysplasia due to a mutation in a TGF-β superfamily member. *Nat. Genet.* **12**, 315–317 (1996).

5. Hayashita-Kinoh, H. *et al.* Membrane-type 5 matrix metalloproteinase is expressed in differentiated neurons and regulates axonal growth. *Cell Growth Differ.* **12**, 573–80 (2001).

6. Folgueras, A. R. *et al.* Metalloproteinase MT5-MMP is an essential modulator of neuro-immune interactions in thermal pain stimulation. *Proc. Natl. Acad. Sci.* **106**, 16451–16456 (2009).

7. Liou, J.-T. *et al.* Spatial and temporal analysis of nociception-related spinal cord matrix metalloproteinase expression in a murine neuropathic pain model. *J. Chinese Med. Assoc.* **76**, 201–210 (2013).

8. Komori, K. *et al.* Absence of mechanical allodynia and Abeta-fiber sprouting after sciatic nerve injury in mice lacking membrane-type 5 matrix metalloproteinase. *FEBS Lett.* **557**, 125–128 (2004).

9. Ahmed, Z., Douglas, M. R., John, G., Berry, M. & Logan, A. AMIGO3 is an NgR1/p75 co-receptor signalling axon growth inhibition in the acute phase of adult central nervous system injury. *PLoS One* **8**, e61878; 10.1371/journal.pone.0061878 (2013).

10. Almutiri, S., Berry, M., Logan, A. & Ahmed, Z. Non-viral-mediated suppression of AMIGO3 promotes disinhibited NT3-mediated regeneration of spinal cord dorsal column axons. *Sci. Rep.* **8**, 10707; 10.1038/s41598-018-29124-z (2018).

11. Altrock, W. D. *et al.* Functional inactivation of a fraction of excitatory synapses in mice deficient for the active zone protein bassoon. *Neuron* **37**, 787–800 (2003).

12. Song, J. *et al.* MicroRNA-488 regulates zinc transporter SLC39A8/ZIP8 during pathogenesis of osteoarthritis. *J. Biomed. Sci.* **20**, 31 (2013).

13. Kim, J.-H. *et al.* Regulation of the catabolic cascade in osteoarthritis by the zinc-ZIP8-MTF1 axis. *Cell* **156**, 730–43 (2014).

14. Haller, G. *et al.* A missense variant in SLC39A8 is associated with severe idiopathic scoliosis. *Nat. Commun.* **9**, 4171; 10.1038/s41467-018-06705-0 (2018).

15. Kolhe, R. *et al.* Gender-specific differential expression of exosomal miRNA in synovial fluid of patients with osteoarthritis. *Sci. Rep.* **7**, 2029; 10.1038/s41598-017-01905-y (2017).

16. Kong, L. *et al.* Interaction between cartilage oligomeric matrix protein and extracellular matrix protein 1 mediates endochondral bone growth. *Matrix Biol.* **29**, 276–286 (2010).

17. Kong, L. *et al.* Extracellular matrix protein 1, a direct targeting molecule of parathyroid hormone-related peptide, negatively regulates chondrogenesis and endochondral ossification via associating with progranulin growth factor. *FASEB J.* **30**, 2741–54 (2016).

18. Deckers, M. M. *et al.* Recombinant human extracellular matrix protein 1 inhibits alkaline phosphatase activity and mineralization of mouse embryonic metatarsals in vitro. *Bone* **28**, 14–20 (2001).

19. Han Z, Ni J, Smits P, Underhill CB, Xie B, Chen Y, et al. Extracellular matrix protein 1 (ECM1) has angiogenic properties and is expressed by breast tumor cells. FASEB J. 2001;15: 988–994. doi:10.1096/fj.99-0934com

20. Fujimoto, N. *et al.* Extracellular matrix protein 1 inhibits the activity of matrix metalloproteinase 9 through high-affinity protein/protein interactions. *Exp. Dermatol.* **15**, 300–307 (2006).

21. Sercu, S. *et al.* Interaction of extracellular matrix protein 1 with extracellular matrix components: ECM1 is a basement membrane protein of the skin. *J. Invest. Dermatol.* **128**, 1397–1408 (2008).

22. Matsuda, A. *et al.* Large-scale identification and characterization of human genes that activate NF-κB and MAPK signaling pathways. *Oncogene* **22**, 3307–3318 (2003).

23. Clark, A. K. *et al.* Inhibition of spinal microglial cathepsin S for the reversal of neuropathic pain. *Proc. Natl. Acad. Sci.* **104**, 10655–10660 (2007).

24. Clark, A. K. & Malcangio, M. Fractalkine/CX3CR1 signaling during neuropathic pain. *Front. Cell. Neurosci.* **8**, 121 (2014).

25. Irie, O. *et al.* Discovery of orally bioavailable cathepsin S inhibitors for the reversal of neuropathic pain. *J. Med. Chem.* **51**, 5502–5505 (2008).

26. Barclay, J. *et al.* Role of the cysteine protease cathepsin S in neuropathic hyperalgesia. *Pain* **130**, 225–234 (2007).

27. Zhang, X., Wu, Z., Hayashi, Y., Okada, R. & Nakanishi, H. Peripheral role of cathepsin S in Th1 cell-dependent transition of nerve injury-induced acute pain to a chronic pain state. *J. Neurosci.* **34**, 3013–3022 (2014).

28. Lai, N.-S. *et al.* Aberrant expression of interleukin-23-regulated miRNAs in T cells from patients with ankylosing spondylitis. *Arthritis Res. Ther.* **20**, 259 (2018).

29. Kramer, P. R. & Wray, S. Novel gene expressed in nasal region influences outgrowth of olfactory axons and migration of luteinizing hormone-releasing hormone (LHRH) neurons. *Genes Dev.* **14**, 1824–34 (2000).

30. Spilker, C., Grochowska, K. M. & Kreutz, M. R. What do we learn from the murine *Jacob/Nsmf* gene knockout for human disease? *Rare Dis.* **4**, e1241361 (2016).

31. Kallenborn-Gerhardt, W., Schröder, K., Geisslinger, G. & Schmidtko, A. NOXious signaling in pain processing. *Pharmacol. Ther.* **137**, 309–317 (2013).

32. Hasan, M. T. *et al.* Role of motor cortex NMDA receptors in learning-dependent synaptic plasticity of behaving mice. *Nat. Commun.* **4**, 2258; 10.1038/ncomms3258 (2013).

33. Lötsch, J. *et al.* Functional genomics of pain in analgesic drug development and therapy. *Pharmacol. Ther.* **139**, 60–70 (2013).

34. Vargha-Khadem, F., Gadian, D. G., Copp, A. & Mishkin, M. FOXP2 and the neuroanatomy of speech and language. *Nat. Rev. Neurosci.* **6**, 131–138 (2005). doi:10.1038/nrn1605

35. Konopka, G. *et al.* Human-specific transcriptional regulation of CNS development genes by FOXP2. *Nature* **462**, 213–217 (2009).
