# Supplementary material for: Analysis of genetically independent phenotypes identifies shared genetic factors associated with chronic musculoskeletal pain conditions
Source: Commun Biol. 2020 Jun 25;3:329. doi: 10.1038/s42003-020-1051-9 (PMC7316754; doi:10.1038/s42003-020-1051-9)
Supplement: Supplementary file 13 — Reporting Summary [file 42003_2020_1051_MOESM13_ESM.pdf]

nature research

Corresponding author(s): Yakov Tsepilov
Yurii Aulchenko

Last updated by author(s): May 22, 2020

Reporting Summary

Nature Research wishes to improve the reproducibility of the work that we publish. This form provides structure for consistency and transparency in reporting. For further information on Nature Research policies, see Authors & Referees and the Editorial Policy Checklist.

Statistics

For all statistical analyses, confirm that the following items are present in the figure legend, table legend, main text, or Methods section.

n/a	Confirmed	
☐	☒	The exact sample size (*n*) for each experimental group/condition, given as a discrete number and unit of measurement
☐	☒	A statement on whether measurements were taken from distinct samples or whether the same sample was measured repeatedly
☐	☒	The statistical test(s) used AND whether they are one- or two-sided<br>*Only common tests should be described solely by name; describe more complex techniques in the Methods section.*
☐	☒	A description of all covariates tested
☐	☒	A description of any assumptions or corrections, such as tests of normality and adjustment for multiple comparisons
☐	☒	A full description of the statistical parameters including central tendency (e.g. means) or other basic estimates (e.g. regression coefficient) AND variation (e.g. standard deviation) or associated estimates of uncertainty (e.g. confidence intervals)
☐	☒	For null hypothesis testing, the test statistic (e.g. *F*, *t*, *r*) with confidence intervals, effect sizes, degrees of freedom and *P* value noted<br>*Give P values as exact values whenever suitable.*
☒	☐	For Bayesian analysis, information on the choice of priors and Markov chain Monte Carlo settings
☒	☐	For hierarchical and complex designs, identification of the appropriate level for tests and full reporting of outcomes
☐	☒	Estimates of effect sizes (e.g. Cohen's *d*, Pearson's *r*), indicating how they were calculated

Our web collection on statistics for biologists contains articles on many of the points above.

Software and code

Policy information about availability of computer code

Data collection	UK Biobank data collection (project #18219); GWAS-MAP platform and data collection (doi: 10.18699/BGRSSB-2018-020)
Data analysis	LDSC (01 Feb 2018), PLINK v1.90b3.31 64-bit (3 Feb 2016), PLINK v2.00a2LM AVX2 Intel (26 Aug 2019), BOLT-LMM v2.3.2, R 3.6.1. All computer code used in this research is available at https://github.com/Sodbo/Pain3_project_code91

For manuscripts utilizing custom algorithms or software that are central to the research but not yet described in published literature, software must be made available to editors/reviewers. We strongly encourage code deposition in a community repository (e.g. GitHub). See the Nature Research guidelines for submitting code & software for further information.

Data

Policy information about availability of data

All manuscripts must include a data availability statement. This statement should provide the following information, where applicable:
- Accession codes, unique identifiers, or web links for publicly available datasets
- A list of figures that have associated raw data
- A description of any restrictions on data availability

UK Biobank data are available upon application. Summary statistics from the GWAS reported in this study are available for download from Zenodo90 under the CC BY 4.0 license.

Field-specific reporting

Please select the one below that is the best fit for your research. If you are not sure, read the appropriate sections before making your selection.

☒ Life sciences ☐ Behavioural & social sciences ☐ Ecological, evolutionary & environmental sciences

For a reference copy of the document with all sections, see nature.com/documents/nr-reporting-summary-flat.pdf

Life sciences study design

All studies must disclose on these points even when the disclosure is negative.

Sample size	Overall, 456,580 individuals from UK BIobank with imputed genotype data and phenotype data were included in the present study. Of these, 265,000 participants of European ancestry (defined by SNP-based principal component analysis) were randomly selected to provide the GWAS discovery cohort. The decision to include only Europeans was based solely on the highest representation of these individuals among the UK Biobank participants.
Data exclusions	We do not define any exclusion criteria except these defined by UK Biobank.
Replication	The replication cohort (N = 191,580) comprised individuals of African (N = 7,541) and South Asian ancestry (Indian, Pakistani, and Bangladeshi; N = 9,208) as well as the remaining European ancestry participants (N = 174,831).
Randomization	Randomization is not relevant to our study design.
Blinding	Blinding is not relevant to our study design.

Reporting for specific materials, systems and methods

We require information from authors about some types of materials, experimental systems and methods used in many studies. Here, indicate whether each material, system or method listed is relevant to your study. If you are not sure if a list item applies to your research, read the appropriate section before selecting a response.

Materials & experimental systems

n/a	Involved in the study	
☒	☐	Antibodies
☒	☐	Eukaryotic cell lines
☒	☐	Palaeontology
☒	☐	Animals and other organisms
☐	☒	Human research participants
☒	☐	Clinical data

Methods

n/a	Involved in the study	
☒	☐	ChIP-seq
☒	☐	Flow cytometry
☒	☐	MRI-based neuroimaging

Human research participants

Policy information about studies involving human research participants

Population characteristics	UK Biobank is a prospective cohort study of over 500,000 individuals from across the United Kingdom http://www.ukbiobank.ac.uk
Recruitment	articipants, aged between 40 and 69, were invited to one of 22 centres across the UK between 2006 and 2010 http://www.ukbiobank.ac.uk
Ethics oversight	All study participants provided written informed consent, and the study was approved by the North West Multi-Centre for Research Ethics Committee (11/NW/0382).

Note that full information on the approval of the study protocol must also be provided in the manuscript.
