# Supplementary material for: Analysis of genetically independent phenotypes identifies shared genetic factors associated with chronic musculoskeletal pain conditions
Source: Commun Biol. 2020 Jun 25;3:329. doi: 10.1038/s42003-020-1051-9 (PMC7316754; doi:10.1038/s42003-020-1051-9)
Supplement: Supplementary file 14 — Description of Additional Supplementary Files [file 42003_2020_1051_MOESM14_ESM.pdf]

Descriptions of Additional Supplementary Files

Supplementary Data 1 (one xlsx file, two sheets, one Table per a sheet):
Supplementary Data 1a. Estimates of SNP-based heritability of chronic pain phenotypes.
Supplementary Data 1b. Description of traits included in the GWAS-MAP database.

Supplementary Data 2. Results of conditional and joint analysis.
Supplementary Data 3. Top loci associated with GIPs at a study-level threshold of statistical significance (P < 1.25e-08).
Supplementary Data 4. Literature data on well-studied SNPs associated with GIP1 and GIP2.
Supplementary Data 5. Gene prioritization based on a literature review.

Supplementary Data 6 (one xlsx file, five sheets, one Table per a sheet):
Supplementary Data 6a. Results of the VEP analysis.
Supplementary Data 6b. Results of the FATHMM-XF analysis for SNPs.
Supplementary Data 6c. Results of the FATHMM-INDEL analysis for indels.
Supplementary Data 6d. Matching alleles with predicted detrimental effects with their effects on GIPs and with lead SNP alleles.
Supplementary Data 6e. SNP set for the VEP and FATHMM analyses.

Supplementary Data 7 (one xlsx file, two sheets, one Table per a sheet):
Supplementary Data 7a. Results of SMR/HEIDI analysis. Searching for pleiotropic effects on GIPs and gene expression. Associations that passed both SMR and HEIDI analyses (P_{SMR} < 3.24e-06 and $P_{HEIDI} \geq 0.01$).
Supplementary Data 7b. Results of SMR/HEIDI analysis. Searching for pleiotropic effects on GIPs and gene expression.

Supplementary Data 8 (one xlsx file, six sheets, one Table per a sheet):
Supplementary Data 8a. GIP1. Results of DEPICT analysis for SNPs with P < 1e-05. Gene prioritization.
Supplementary Data 9b. GIP1. Results of DEPICT analysis for SNPs with P < 5e-08. Gene prioritization.
Supplementary Data 8c. GIP1. Results of DEPICT analysis for SNPs with P < 1e-05. Tissue enrichment analysis.
Supplementary Data 8d. GIP1. Results of DEPICT analysis for SNPs with P < 5e-08. Tissue enrichment analysis.
Supplementary Data 8e. GIP1. Results of DEPICT analysis for SNPs with P < 1e-05. Gene set enrichment analysis.
Supplementary Data 8f. GIP1. Results of DEPICT analysis for SNPs with P < 5e-08. Gene set enrichment analysis.

Supplementary Data 9. Statistically significant results of gene set enrichment analysis conducted using the FUMA platform.
Supplementary Data 10. Results of SMR/HEIDI analysis. Searching for pleiotropic effects on GIPs and other complex traits. Associations that passed both SMR and HEIDI analyses (P_{SMR} < 3.71e-06 and $P_{HEIDI} \geq 0.01$).

Supplementary Data 11 (one xlsx file, four sheets, one Table per a sheet):
Supplementary Data 11a. Genetic correlations between GIP1 and human complex traits.
Supplementary Data 11b. Genetic correlations between GIP2 and human complex traits.
Supplementary Data 11c. Genetic correlations between GIP3 and human complex traits.
Supplementary Table 11d. Genetic correlations between GIP4 and human complex traits.
